# Identification of Distinct, Quantitative Pattern Classes from Emergent Tissue-Scale hiPSC Bioelectric Properties

**DOI:** 10.3390/cells13131136

**Published:** 2024-07-02

**Authors:** Dennis Andre Norfleet, Anja J. Melendez, Caroline Alting, Siya Kannan, Arina A. Nikitina, Raquel Caldeira Botelho, Bo Yang, Melissa L. Kemp

**Affiliations:** 1 The Wallace H. Coulter Department of Biomedical Engineering, Georgia Institute of Technology and Emory University, 950 Atlantic Dr. NW, Atlanta, GA 30332, USA; dnorfleet7@gatech.edu (D.A.N.);; 2 School of Biological Sciences, Georgia Institute of Technology, Atlanta, GA 30332, USA; 3 Neuroscience Research Institute, University of California Santa Barbara, Santa Barbara, CA 931016, USA; 4 George W. Woodruff School of Mechanical Engineering, Georgia Institute of Technology, Atlanta, GA 30332, USA

**Keywords:** image pattern recognition, bioelectricity, systems biology, emergence, voltage membrane potential, cell-cell communication, computational modeling, pluripotent stem cells

## Abstract

Bioelectric signals possess the ability to robustly control and manipulate patterning during embryogenesis and tissue-level regeneration. Endogenous local and global electric fields function as a spatial ‘pre-pattern’, controlling cell fates and tissue-scale anatomical boundaries; however, the mechanisms facilitating these robust multiscale outcomes are poorly characterized. Computational modeling addresses the need to predict in vitro patterning behavior and further elucidate the roles of cellular bioelectric signaling components in patterning outcomes. Here, we modified a previously designed image pattern recognition algorithm to distinguish unique spatial features of simulated non-excitable bioelectric patterns under distinct cell culture conditions. This algorithm was applied to comparisons between simulated patterns and experimental microscopy images of membrane potential (V_mem_) across cultured human iPSC colonies. Furthermore, we extended the prediction to a novel co-culture condition in which cell sub-populations possessing different ionic fluxes were simulated; the defining spatial features were recapitulated in vitro with genetically modified colonies. These results collectively inform strategies for modeling multiscale spatial characteristics that emerge in multicellular systems, characterizing the molecular contributions to heterogeneity of membrane potential in non-excitable cells, and enabling downstream engineered bioelectrical tissue design.

## 1. Introduction

The advent of induced pluripotent stem cell technologies has facilitated studies of developmental trajectories and multicellular system processes. Populations of human induced pluripotent stem cells (hiPSCs) represents ideal systems to study and probe development, mimicking embryonic tissue in the ability to differentiate to the three extraembryonic lineages and self-organize into organoid structures in a robust manner [1,2]. Understanding the interactions between intrinsic biochemical, bioelectric, and biomechanical cues driving multicellular emergent features would enable directed, novel morphogenic trajectories into novel engineered tissues, given the proper tools and biotechnologies [3]. Multi-scale computational model simulations are well-suited for uncoupling roles of specific system components from interactions with other larger- or smaller-scale system components [4,5] that would be difficult, if not infeasible, to perform experimentally. Previous computational studies have elucidated individual biochemical and biomechanical spatiotemporal regulators of development through computational modeling [6,7,8,9].

While these modeling and experimental studies have successfully characterized individual aspects of biochemical and biomechanical cues [10,11], analogous hiPSC modeling/experimental studies of bioelectric signals have not been performed. Ion channels, gap junctions, and ion pumps coordinate activities to create and affect cellular membrane voltage potential (V_mem_) and steady-state V_mem_ patterns in interconnected cell clusters of non-excitable cells. Embryonic channelopathies lead to morphological deformities in lower-level complexity organisms, such as Drosophila, and X. Laevis, and higher-level complexity mammals, such as chick, rodent, and human embryos [12,13]. Computational modeling has already helped to elucidate the role of specific ion channels in the emergent bioelectric pre-patterns of other organisms. A study done on multi-scale bioelectric controls of development in lower-complexity species, *X. laevis*, found that spatial long-range V_mem_ gradients were critical ‘pre-patterns’ for neural crest morphogenesis in embryogenesis, and the authors established interconnected roles for bioelectric machinery that was responsible for creating and maintaining that pattern [14]. The authors integrated quantitative cell electrophysiological and functional data to parameterize the bioelectric computational model to their specific in this developmental context and validated the criticality of a particular voltage-gated K^+^ channel isoform, HCN2. In related work, computational simulations of non-excitable cells highlighted the critical role gap junctions hold in intercellular coupling and suggested potential mechanisms by which bioelectric state information propagates from the single- to multicellular scale [4,15,16]. The disruption of intercellular communication has been shown to disrupt morphological outcomes in various multicellular systems [5,17] and this perspective of gap junctions as information processing conduits has received experimental support [18]. While the aforementioned studies were instrumental in setting a precedent for studying bioelectric signaling in multicellular modeling, subsequent extension to the human developmental processes has not been performed due to the challenges of parameterizing human iPSC ionic flux regulation on more than one level of spatial scale (cellular or subcellular); therefore, previous characterizations of human electrophysiology have either focused on the molecular scale of ion channel or cellular scale of whole cell ionic current [19,20]. 

The lack of corresponding bioelectric characterization and need for a multi-scale understanding of bioelectric system emergent interactions in hiPSCs motivated us to develop a multi-scale modeling characterization of bioelectric patterning. A better understanding of how these signals propagate in human bioelectric multicellular systems may enable engineering of specific cell types to occupy specified regions within a cluster and enhance their desired functional capacity in cell transplants in cell manufacturing and regenerative medicine applications. 

**Figure 1:**
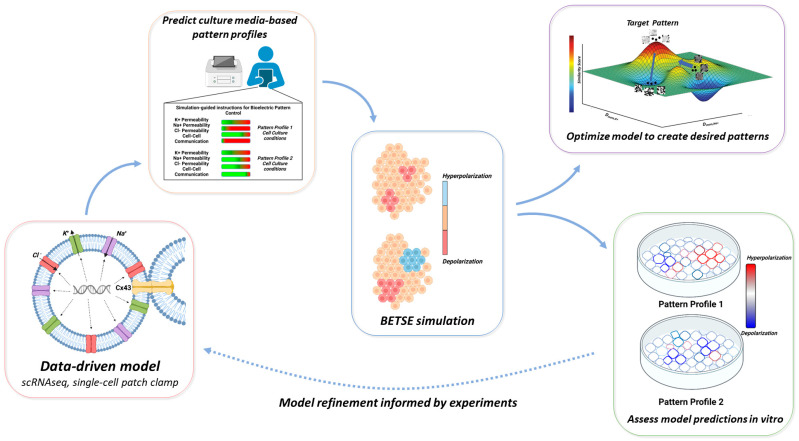
A broad overview of the aspects of the hiPSC bioelectric system covered in this study. First, we describe the initial model definition procedure, in which electrophysiologic and transcriptomic, and literature data was leveraged for describing hiPSC colonies (**bottom, left**). From this model, culturing conditions and perturbations are replicated in simulation configuration files and multiple output pattern images produced (**center**). These patterns are converted into compact formula representation, then compared to (**bottom**, **right**) experimentally produced bioelectric pattern profiles and (**top**, **right**) pattern profiles produced from novel optimized parameter combinations. These outcomes also collectively enable model improvements, based on discrepancies pattern profile similarity between simulated and experimental images.

To holistically understand the influence of bioelectric system component perturbations on resulting patterns, we parameterized a previously developed multi-physics simulation engine, Bioelectric Tissue Simulation Engine (BETSE) [21,22] to predict human induced pluripotent stem cell (hiPSC) bioelectric patterns (Figure 1). The mechanistic model is a bottom-up scheme that utilizes known basic relationships between ion flux, V_mem_, and cell-cell communication to form predictions on the 2-D bioelectric patterns that form from the intrinsic interplay between system components and the external environment under differing monolayer cell culture conditions ranging from pharmacological inhibition, cell culture media alterations, and CRISPRi. We trained machine learning-based algorithms to identify unique ‘rules’ that represent spatial features distinct to each bioelectric pattern condition. Quantitative image similarity comparisons showed that computational simulations were able to recapitulate distinctions between emergent bioelectric patterns under various conditions. These predictions performed under a variety of simulated contexts support the approach of this bottom-up modeling characterization for investigating multicellular bioelectrical phenomenon.

## 2. Materials and Methods

### 2.1. Contribution of Active Ionic Transport to Steady-State V_mem_ Maintenance

Fluorescence intensity measurements were recorded in unaltered mTeSR (Stemcell Technologies, Vancouver, BC, Canada) control samples and PFA-fixed (4%) iPSCs in mTeSR. Ouabain (Sigma-Aldrich, St. Louis, MO, USA), which binds to the extracellular domain of the Na^+^/K^+^-ATPase α3 helix to inhibit the enzyme’s ionic transport activity, was then pipetted into the control sample FluoroDish. The change in fluorescence intensity that accompanies the ouabain addition was compared against those of the control and depolarized iPSC sample measurements after post-background correction. This allowed quantitative estimation of active transmembrane ionic flux contribution to the establishment and creation of resting V_mem_ in iPSCs and is used in the model parameterization of active ion pump ion flux dynamics in the bioelectric model.

### 2.2. WTC11 hiPSC Cell Culture

The human induced pluripotent stem cell line (WTC11, Coriell, Camden, NJ, USA) was maintained between passages 50 and 70 in GFR-Matrigel coated 6-well plates (Corning, Corning, NY, USA) in mTeSR PLUS (Stemcell Technologies, Canada) media was used to maintain cells. Ccells were fed daily until passage (3–4 days), when they were dissociated using Accutase solution and subsequently reseeded seeded at 90,000 cells per well (~9400 cells/cm^2^) or into FluoroDish culture plates (WPI INC., Sarasota, FL, USA) at 100–110,000 cells per well (10,400–11,500 cells/cm^2^). The cell cultures were incubated at 37 °C and 5% CO_2_.

### 2.3. Calibration of DiBAC to Cellular V_mem_

Cells were grown to 75–90% confluency. Cells were subsequently washed with mTeSR and replaced with a DiBAC-infused media solution and incubated in the dark for 30 min. The control FluoroDish received 0.5 μM DiBAC and PFA-fixed FluoroDishes received concentrations of 0.5, 1, 1.5, and 2 μM. Multiple ROIs (n ≥ 3) were captured for each plate/DiBAC concentration. ROI confocal images were subsequently exported as TIFF files for downstream image correction and intensity analysis. The method, previously utilized by [23], assumed a direct relationship between V_mem_ and fluorescence intensity given the equilibrium Nernstian charge distribution assumptions of equal intra- and extracellular free dye concentration (DiBAC_4_(3)).

### 2.4. Whole-Cell Electrophysiological Patch Clamp of WTC11 hiPSCs

To assess the voltage dependence of transmembrane ionic current density, the cells were seeded on a coverslip, bathed with saline buffer solution in the patch clamp chamber, and patched with microelectrode pipettes for current or voltage measurement recordings. The whole cell configuration between the pipette and the cell allows the control of extracellular and intracellular ionic concentrations during an experiment. All recordings were performed in a HEPES buffer solution consisting of (in mM): 120 mM NaCl, 3 mM KCl, 1.8 mM NaH_2_PO_4_, 26 mM NaHCO_3_, 2 mM CaCl_2_, 2 mM MgCl_2_ and 10 mM dextrose, pH adjusted to 7.4 with NaOH. The internal solution contained (in mM): 120 mM K-gluconate, 10 mM HEPES, 10 mM KCl, 0.2 mM EGTA, 2 mM MgATP, 0.3 mM NaGTP. The patched cell was subjected to a step voltage protocol in which the cell-attached micropipette was increased in a stepwise manner to varying V_mem_ magnitudes from −70 to 20 mV in 10 mV increments. The measured single-cell current magnitude at each step voltage value was then plotted on a characteristic current-voltage (I–V) graph, which enabled downstream parameterization of the bioelectric model for determination of single-cell V_mem_-dependent transmembrane ionic flux magnitudes.

### 2.5. GJA1-Modified hiPSC Cell Culture and Experiment Preparation

The LBC2 cell line (parental iPSC) was used as a control and the LBC2-GJA1-CRISPRi cell line (both gifts of Todd McDevitt, Gladstone Institute, San Francisco, CA, USA) were used to investigate connexin 43 (Cx43; GJA1) expression in stem cell colonies. The LBC2-GJA1-CRISPRi cells were initially thawed and maintained in Doxycycline-treated mTeSR culture media for 7 days to achieve full GJA1 dCas9 silencing, then subsequently transferred to FluoroDishes for experiment preparation. Both doxycycline-treated and untreated GJA1-CRISPRi cultures were maintained in FluoroDishes (n = 3 dishes per condition) and imaged using the same DiBAC-mTeSR media solution as described above. Untreated GJA1-CRISPRi images were compared to control WTC11 DiBAC image networks downstream for class similarity comparison.

### 2.6. Gap Junction Pharmacological Molecule Inhibition

WTC11 hiPS cells were seeded at an initial density of 90,000–110,000 cells per FluoroDish and grown to 80–90% confluency. Cells were then rinsed with mTeSR and subsequently incubated fed with a DiBAC-infused mTeSR solution (1 μM) then were incubated in the dark for 30 min prior to imaging at 37 °C. PerkinElmer Spinning Disk (Waltham, MA, USA) and Nikon W1 Spinning Disk Confocal microscopes (Melville, NY, USA) were used to captured DiBAC stained cell colony image samples in the both prior to and 10 min following 10 or 60 μM 18β-Glycyrrhetinic acid (Sigma-Aldrich, St. Louis, MO, USA) media addition. This time interval was chosen to capture cell colonies in the period where the drug molecule maximally exerts its effects on cell-cell current, as previously shown by [24]. After the experiment conclusion, images were exported in TIFF format to ImageJ (version 1.54a) for post-processing and analysis.

### 2.7. K^+^ Supplementation DiBAC Experiment

WTC11 hiPS cells were seeded at an initial density of 90,000–110,000 cells per FluoroDish and grown to 80–90% confluency. Cells were aspirated and rinsed with dPBS before DiBAC-mTeSR application and incubation. Then, cells were placed in a modified K^+^-supplemented DiBAC-mTeSR solution and subsequently incubated in the dark prior to fluorescent confocal imaging. The modified media preparation involved initial DiBAC serial dilution to 100× working concentration, as usual. However, a separate 100 mM K^+^-supplemented mTeSR solution was prepared during the second serial dilution step. Subsequent DiBAC-mTeSR solution dilution to the final culture concentration (1 μM) was combined with the previously prepared 100 mM K^+^-supplemented mTeSR stock solution in proportion to the final imaging concentrations of 10 and 20 mM [K^+^]. Cells were incubated in the dark for 30–60 min prior to imaging. All sample images were collected on a Nikon W1 Spinning Disk Confocal Microscope at 60× magnification. Sample images were obtained at different ROIs throughout the FluoroDish and stored for downstream post-processing and image analysis.

### 2.8. Experimental Image Transformation, Segmentation, and Analysis

To convert the DiBAC experiment output images to grayscale bioelectric cell pattern representations, we initially performed background and noise correction on raw image data in ImageJ. Corrected images were subsequently exported to CellProfiler, where they underwent a fluorescence intensity-based transformation procedure. The cells were identified as objects within each image, based on the fluorescent labeling of the cell lipid membrane bilayer. Specifically, a custom script used the roughly circular cell fluorescent border to mark the boundary of a particular cell object and the enclosed region represented the cell interior. Next, cell objects were expanded until touching adjacent cell object borders and calculations were performed over the encompassed cell object region and normalized by cell circumference to obtain a normalized cell fluorescence intensity value; this value represented the relative quantitative cell V_mem_ observation. A grayscale colormap was applied to the resultant image to identify cell V_mem_ state with a grayscale color shade, synonymous to the way BETSE model output images visually define cellular V_mem_. Finally, transformed grayscale images were autoscaled to image minimum and maximum pixel intensities and exported for downstream quad-tree decomposition and pattern analysis.

Raw output images were exported as ‘.tiff’ files into ImageJ, where images were pre-processed prior to transformation in CellProfiler. For each FluroDish used to image a hiPS cluster ROI, an associated image ROI containing no cells was recorded and denoted as the ‘flatfield image’ [25]; this image’s fluorescence intensity was subtracted from that of the Raw output ROI image. The processed images were then exported to CellProfiler for further processing. CellProfiler (http://cellprofiler.org/) (accessed on 11 December 2023) was utilized to transform experimental DiBAC fluorescent images into a grayscale recapitulation. The first step employed an image smoothing and closing filter to eliminate ‘salt-and-pepper noise’ within the image; images were then subjected to a cell masking procedure in which the script identified the DiBAC fluorescent pixels as the cell border. The image space (area) pertaining to the region enclosed by the identified border was used to identify the cell itself and associated morphology in the ‘EnhanceOrSupressFeatures’ module; another filer ‘ExpandOrShrinkObjects’ expanded these morphological borders expanded to create adjacent boundaries between cells; this was done to ensure the least amount of bias when comparing pattern images across domains to the computationally rendered images. Next, infinitesimal fluorescence intensities (FIs) measured over the whole identified cell ‘object’ border (membrane) area and divided by cell area; the resulting single value produced represented an averaged object FI value per cell. This FI served as a directly proportional qualitative metric to average cell V_mem_. This method of initial transformation was done to ensure qualitative proximity to BETSE output images. Furthermore, BETSE utilizes a similar strategy to calculate the single numerical value used to represent cell V_mem_ state in output cluster image ROIs. The resultant grayscale images are exported to Matlab for further image processing. The images were initially loaded as matrices, in which colormaps numerically represented greyscale fluorescence intensities or qualitative model cell V_mem_ estimations. An RGB colormap is customized to have 3 discrete bins that separate colors and their respective intensities. This bin breakdown was subsequently used for both in vitro and in silico images inputs to be utilized in downstream image quad-tree decomposition and analysis.

### 2.9. Immunocytohistochemistry and Imaging

LBC2 and LBC2-GJA1 hiPSCs were grown to 85–90% confluency on FluoroDishes, then fixed for 15 min at room temperature with 4% paraformaldehyde (Electron Microscopy Sciences, Hatfield, PA, USA). Fixed colonies were subsequently washed 2 times with dPBS. Colonies were then blocked and permeabilized in 1× PBS and 0.3% Triton X-100 (Sigma-Aldrich) and 5% Normal Donkey Serum (Sigma-Aldrich) for 60 min at room temperature. hiPSC colonies were subsequently incubated with primary antibody Cx43-CST (Sigma-Aldrich) in a 1× PBS blocking buffer solution with 0.3% Triton X overnight at 4 °C. The following day cells were washed with PBS 2 times, then were incubated with secondary antibody solution of goat anti-rabbit Alexa Fluor 647 (Thermo Fisher Scientific, 1:200, Eugene, OR, USA) in antibody dilution buffer for 60 min in the dark at room temperature. The colonies were then counterstained with Hoeshst 33342 for 5 min before samples were transported to the confocal microscope (Nikon W1 Spinning Disk Microscope; Melville, NY, USA) for imaging. All images were taken at 60× magnification.

### 2.10. Single-Cell RNA Sequencing of hiPSCs

Undifferentiated WTC11 hiPSCs were maintained on GFR-Matrigel and cultured in mTeSR culture media, as previously described, and subsequently cryogenically preserved until single cell RNA sequencing assay preparation. On the experiment day, cells were thawed and initially combined with molecular labels. First, cells were individually profiled by addition of poly(dT) primers in a solution mix. The cell suspension was composed of cells, partitioning oil, and primers, enabled generation of barcode-tagged cell droplets. These droplets were then loaded on 10× Genomics Single Cell 3′ Chips to generate single-cell gel beads in emulsion (GEMs). Immediately following GEM production the gel bead coat was dissolved, allowing release of lysed cell contents and identifying molecular barcode label. These products underwent subsequent cDNA amplification via reverse transcription and purification. The amplified genetic products were used with each sample’s unique barcode label to construct sample-specific complementary DNA (cDNA) libraries in the Chromium instrument. The resulting barcoded libraries were then multiplexed and subjected to a standard NGS short-read sequencing protocol; the output data from these sequencing runs were cell-specific transcriptomic expression profiles.

### 2.11. Processing Raw Single-Cell RNA Sequencing Data

Processing of sample scRNA-Seq data was conducted using Cell Ranger software by the 10× Genomics company (https://www.10xgenomics.com/, (accessed 17 January 2020)). Raw base call (BCL) files generated from the Illumina sequencer were first demultiplexed into sample specific FASTQ files by utilizing the cellranger mfastq pipeline. Then, FASTQ files were processed through the cellranger count pipeline for each cell culture sample. More specifically, cellranger count used the STAR RNA-Seq aligner tool [26] to align cDNA measured reads to the human reference genome, hg19. Then, aligned reads were checked for valid cell barcodes and unique molecular identifiers (UMIs) and filtered. Finally calculated UMI reads were normalized in Seurat [27] and exported as sample-specific matrices containing expression values as counts per million (CPM), or transcripts per million (TPM). UMAP plots were generated in accordance with the Seurat non-linear dimensional analysis pipeline [27]. All parameters used were set to their default values.

### 2.12. Computational Model (BETSE)

The model is designed to simulate bioelectrical patterns that arise from single-cell bioelectric dynamic parameters implemented in a multicellular colony. Modeling simulations are performed in the computational platform, Bio-Electric Tissue Simulation Engine (BETSE) [28,29]. The details of the platform architecture are covered in Appendix A. Ion fluxes are generated by transmembrane transport via ion channels and active pumps. The combined action of fluxes shown above leads to changes in individual cellular spatial ion concentrations. The fundamental charge property of the modeled ions assumes an accompanying change in spatial intracellular charge density and ionic current density ($\vec{J}$).

The model assumes “passive electrodiffusive mass transport” that can utilize three distinct pathways: (1) *transmembrane:* via intra- and extracellular spaces across the plasma membrane; (2) *intercellular (GJ):* between cellular spaces via gap junctions; and (3) *extracellular:* within the global environment and extracellular spaces. BETSE mathematical strategy incorporates electrochemical mass transport biophysical characterizations to describe how ions move within a multicellular cluster. Passive electrodiffusive ionic flux is mathematically characterized by the Goldman–Hodgkin–Katz Flux equation (GHK Flux equation), where electric field-mediated flux between local intra- and extracellular spaces dictates spatiotemporal ion dynamics. Changes in concentration are determined by assuming the concentration change in an ion *i* depends on the divergence of the net ($\Phi_{Total,\;i}$) sum of all fluxes of the ion entering or changing in a particular region of the space (i.e., cells or environment).

(1) $$I_{Total}=\sum Fz_{i}\Phi_{i}=I_{pump}+I_{{Na}^{+}}+I_{K^{+}}+I_{{Cl}^{-}}$$

This characterization of whole–cell ionic current as a summation of individual ionic contributors has been explored and experimentally validated in a study from [30]. This assumed structure suggests that experiments aimed at specific knockdown/inhibition of the involved ionic currents can elucidate the magnitude of each component to whole-cell current dynamics at resting V_mem_ and other physiologically relevant V_memS_ (−70 to +20 mV). This relation importantly allows for precise and easy transformation between experimentally measured and in silico ion currents. Further details of parameter assignment and mathematical formulation are detailed in Appendix A.

### 2.13. Computational Image Analysis

Simulation-generated output pattern images were cropped to exclude x-/y-axes and scale bars, and to only include square cell cluster ROIs. Additionally, auto-scaled colormap settings were chosen for each generated pattern image; this was done to ensure a standardized format between the in silico and in vitro domains and to elucidate distinct local and global pattern features that may be otherwise lost. Simulated image sets were stored in directories labeled in accordance with the simulated cell culture condition for downstream quad-tree decomposition, pattern identification, and quantitative pattern similarity comparison.

### 2.14. Quad-Tree Image Representation and Tree Spatial Superposition Logic

We applied a previously developed tree spatial temporal logic (TSSL) algorithm [31] to quantitatively describe/define computational and experimentally obtained multicellular patterning This is essentially the mechanism by which the respective patterns are quantitatively described/defined by. The quantitative valuation of an input image defines how each node (corresponding to a unique pattern) presents itself with respect to the remaining ‘image space’ (corresponding to the global cell cluster). This methodology yields similarity scores between pattern classes which are reported as quantitative values throughout the study results. The description of the image analysis and scoring are provided in Appendix B.

### 2.15. Particle Swarm Optimization

The BETSE simulation input parameters D_mem,K^+^_, D_mem,Na^+^_, D_mem,Cl^−^_, and $\beta_{GJ}^{o}$, play critical roles in determining global and local features of resulting output pattern images. If the parameter set is defined as $p\in \Omega \subset R^{N_{p}}$, where Ω represents a possible parameter range or set of parameter ranges and $N_{p}$ represented the total number of adjusted parameters. Images from culture condition-specific BETSE image sets (numbered 1, …, T) were processed through the TSSL algorithm and produced corresponding individual quadtrees and roots, Q[t] and $v_{0}[t]$, respectively, for each image within the set. The target pattern specification against which simulated images are compared is denoted as $\Phi_{Pattern}$; the quantified relationship for a set of simulated images resulting from parameterization values p is defined with the following equation:

(2) $$S\left(p\right)={max}_{0\leq t\leq T}\rho (\Phi_{Pattern},\;v_{0}[t])$$

The similarity score was denoted by ρ and $S\left(p\right)$ was denoted as a random variable, and used to track simulation images possessing positive valuations, which indicated pattern presence. Subsequently, the objective is defined as maximizing the score $S\left(p\right)$ for a parameterization $p^{*}$.

(3) $$p^{*}={argmax}_{p\in \Omega }E\left(S\left(p\right)\right)$$

This algorithm searches for the parameters that, when averaged together, produced the maximum similarity score to the target pattern classifier. To accomplish this, the algorithm utilized average scores from *n* simulations to account for noise in similarity scoring and simulation stochastic initialization. For *n* simulations, the following mean similarity score expression was utilized:

(4) $$E\left(S\left(p\right)\right)\approx \tilde{S}\left(p\right)=\left(\frac{1}{n}\right)*\sum_{i=1}^{n}S_{i}\left(p\right)$$

where $S_{i}\left(p\right)$ represents the similarity score for parameter set p of the $i^{th}$ simulation. Similar to [6], we ran 3 simulations for each potential parameter set (n = 3). Particle swarm optimization was chosen as the algorithm of choice to address the unknown structure of the solution space. It is a heuristic technique that is especially well-suited to explore irregular search spaces to find local maxima or minima.

To summarize the algorithm: a set of particles is initialized at random points in parameter space, where each particle’s position and velocity were denoted by $z_{i}\in \Omega$ and $z_{i}^{\prime }$, respectively. The particle’s position represented a value within the allowable parameter bounds and the particle’s velocity represented a search direction from the currently implemented solution; the particle’s position and velocity were then updated in accordance with the follow expressions after each simulated iteration:

(5) $$z_{i}^{\prime }\leftarrow Wz_{i}+\eta \left(r_{p}\right)\left(z_{i}^{*}-z_{i}\right)+\eta (r_{g})(z^{*}-z_{i})$$

$$z_{i}\leftarrow z_{i}+z_{i}^{\prime },$$

where $\eta \left(r_{i}\right)$ is a random number selected from distribution [0, $r_{i}$], and the parameters $W\in R,\;r_{p},r_{g}$ are tuned by the user. This process is iterated until the similarity score error between the target pattern and input parameter set was less than 1 × 10^−2^, or the similarity score was maximized over the allowable parameter search space. In the case that BETSE produced positive similarity scores or negative similarity scores near zero for $\tilde{S}\left(p^{*}\right)$, the associated parameter set was called the optimized parameters.

### 2.16. Software Availability

Statistical significance amongst normalized and raw similarity scores was performed using Welch’s *t*-tests with SciPy Python library (modules: numpy, scipy). Quantification of similarity scores between input images and target classifiers was calculated using custom Matlab and Python code. All custom code can be accessed from: https://github.com/andre-norfleet/BETSE-repo (accessed 1 December 2022).

## 3. Results

### 3.1. Electrophysiologic and Bioinformatic Data Enable hiPSC-Specific Bioelectric Modeling

We initiated our model development by focusing on the lowest-scale components of the multicellular bioelectric system individually (Figure 1). In non-excitable stem cells, the critical determinants of cellular resting V_mem_ are ion channels, gap junctions, and ion pumps; multiple studies [32,33,34] have proven that these core bioelectric system components must coordinate activities to create and maintain cell resting V_mem_ state [30,35] and multicellular bioelectric patterns profiles during organogenesis [14,35] and embryogenesis [17,36,37]. Therefore, we hypothesized that this conserved single-cell bioelectric system relationship would also extend to multicellular system dynamics in a multiscale manner via intercellular conduits. Specifically, single-cell states have been shown to directly influence the V_mem_ of neighboring cells via gap junctions, thereby influencing the development of ‘long-range’ voltage gradients across multicellular clusters or tissues [35,38,39], both in in vitro and in vivo contextual domains.

**Figure 2:**
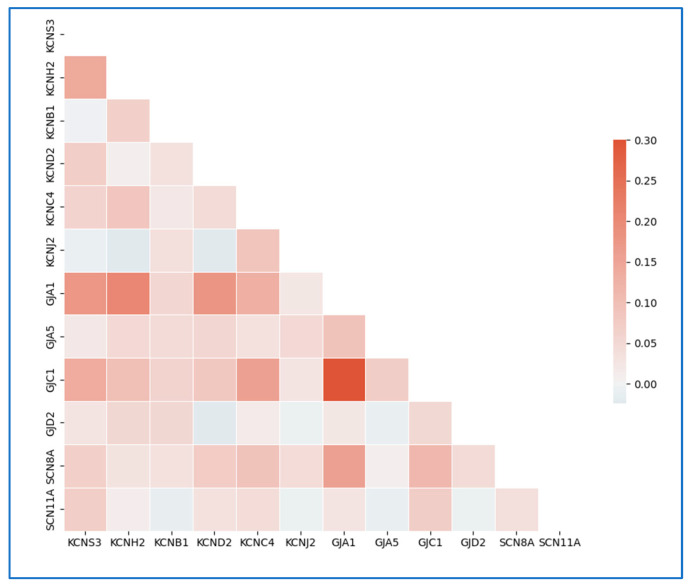
Correlation matrix of single cell transcriptomics for ion channels and connexins expressed in unmodified WTC-11 human iPSC cells bioelectrically characterized in this study. Visualization of single cell expression heterogeneity is provided in Figures S3–S5 (n = 1206 cells).

Single-cell model bioelectric component dynamics were constructed piecewise from the available literature concerning each component’s V_mem_-dependent ionic transport properties [40]. Previous studies have utilized whole-cell patch clamp voltage-clamp assay measurements to identify and quantitatively estimate individual ionic currents elicited from the cell [30,40]. We extended this logic to our single-cell hiPSC model building effort. At the single-cell level, the computational recapitulation of the whole-cell step voltage protocol should accurately reflect the whole-cell currents elicited at different resting V_mem_ values (Figure S6); mathematically, this value should also be equivalent to the net flux from the collective subcellular system components [30]. The Na^+^ and Cl^−^ ionic currents are defined with passive diffusion-based voltage-dependent behaviors, but the K^+^ ion flux is modeled to fit the missing ion magnitude that would create alignment between the BETSE and experimentally produced hiPSC current-voltage (I-V) curve via linear regression. We justified this assumption because K^+^ voltage-gated ion channels showed physiologically relevant levels of expression with respect to the other main ionic components’ respective channel expression levels (Figure 2 and Figures S3 and S5). The cells form dynamically interacting networks due to the ability to form cell-cell gap junction pathways; therefore, we investigated the most abundant and functional gap junction expressed in human embryonic development, Connexin 43 (Cx43; GJA1) [24] (Figure 2 and Figure S4). Correlations in single cell expression between GJA1 and K^+^ channels KCNS3, KCNH2, and KCND2 suggest that under resting conditions, iPSCs will have variability in transmembrane ion currents at the single cell level (Figure 2). While this data was not directly used to model the heterogeneity of resting iPSC V_mem_, it justifies our use of a range of diffusion values as described in the model optimization methods. We estimated hiPSC intercellular ion transport dynamics and their voltage dependence by mathematically converting empirical relationships defined dual-cell patch clamp characterization data [41] empirical transformation to model gap junction maximum junctional conductance and voltage sensitivity parameters. We fit Na^+^/K^+^-ATPase maximum reaction rates to published conductance values [29,42]. Finally, we defined the model external conditions to mimic the ionic composition of mTeSR cell culture media, completing the environmental, electrophysiologic, and transcriptomic characterization of the ‘hiPSC-specific’ bioelectric environment. Furthermore, this bioelectric system condition will serve as the “baseline” control simulated cell culture condition for the remainder of this study.

### 3.2. Single-Cell Bioelectric Dynamics Enable hiPSC Multicellular Pattern Prediction under Varying Cell Culture Conditions

**Figure 3:**
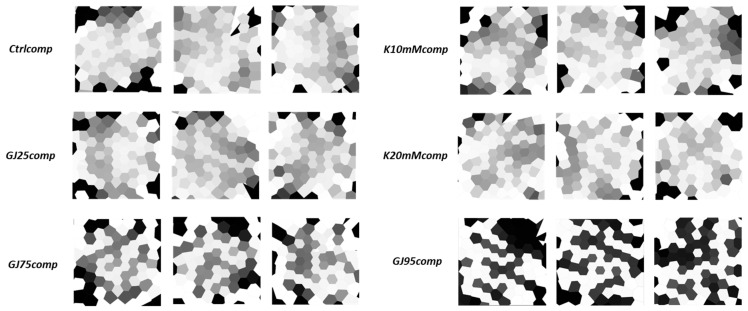
Sample output images of BETSE simulations under the six conditions. Each cell is represented as an irregularly shaped grayscale masked object, and the degree of darkness indicates relative V_mem_ state, or more specifically depolarization with respect to other cells in the cluster. Refer to Table 1 for more details on conditions. Condition nomenclature: ‘Ctrlcomp’ = Un-modified culture media, simulated patterns; ‘GJ25comp’ = Weak inhibition of gap junction cell-cell communication, simulated patterns; ‘GJ75comp’ = Strong inhibition of gap junction cell-cell communication, simulated patterns; ‘GJ95comp’ = Almost complete inhibition of gap junction cell-cell communication, simulated patterns; ‘K10mMcomp’ = 10 mM KCl culture media supplement, simulated patterns; ‘K20mMcomp’ = 20 mM KCl culture media supplement, simulated patterns.

We specified conditions that are both amenable to simulation and recapitulation in vitro culture in that they cause changes in the internal or external composition of the hiPSC bioelectric system. Below, we describe the simulation of two major classes of perturbing the bioelectric properties of the iPSC colonies (i) attenuating connexin-based ion exchange that will be tested experimentally by CRISPRi silencing or small molecule inhibitor, and (ii) addition of K^+^ salt at different doses to impact currents through K^+^ ion channels. Once culture condition-specific single-cell, inter-cellular, and environmental hiPSC parameters were properly defined in the model setup, simulations were run iteratively, and output bioelectric pattern images were saved for each condition (≥199 images per culture condition) (Figure 3).

To obtain a mathematical representation of partial cell-cell communication suppression for the first class of perturbations, we related the literature-associated drug effects on connexin conductance to the most similar in silico parameter governing gap junction conductance. 18-β-Glycyrrhetinic Acid (18-β-GA), a small biomolecule inhibitor, has been reported [32,43,44,45] to partially inhibit transjunctional ion flux between neighboring cells, and this relationship spans physiological values of transjunctional V_mem_ (−60 to +60 mV). Importantly, these patch-clamp characterizations indicate that transjunctional gating voltage sensitivity remains relatively unaffected by the compound. 18-β-GA empirically shifts the cell-cell current-voltage relationship to lower conductance values, consistent with its mechanism of action in which binding of the molecule uncouples gap junction functions through partial or complete disassembly [45].

To model conditions of gap junction inhibition by 18-β-GA, we modified the simulation parameter controlling intercellular ion flux magnitude ($\beta_{GJ}^{0}$) but did not alter V_mem_-sensitive gating parameters. We varied $\beta_{GJ}^{0}$ between the completely open (1 × 10^−7^) and closed (1 × 10^−9^) values. This effectively decreased the effective connexin pore radius connecting cells, which effectively mimics the phosphorylation effect of β-GA on gap junction activity. Two distinct gap junction block classes were simulated in BETSE which were distinguished by the respective relative strengths of gap junction conductance inhibition, namely 25% and 75% gap junction intercell conductance decrease, denoted by ‘GJ25comp’ and ‘GJ75comp’ respectively. As an alternative attenuation of connexin-based flow, simulations of GJA1 CRISPRi modified colonies were achieved by establishing a simulation pattern class associated with a 95% decrease in gap junction intercellular communication capacity, ‘GJ95comp’, based on our observation from transcriptomic data that the majority of hiPSC connexin expression consists of the Cx43 (GJA1) isoform. Retention of 5% GJC flux reflected our modeling assumption that slight expression of GJC1 could compensate for GJA1 silencing (Figure S4). Under simulated conditions of attenuated GJC flux, we observed a discernable trend of bioelectrical cellular uncoupling that increased with gap junction inhibition (Figure 3) such that at the largest decrease in GJC flux yields isoelectric “islands”. This is a striking feature of our models that can be used for machine learning-based pattern classification.

For the second class of simulated conditions, we hypothesized that heterogeneity in Kv7.2 (KCNQ2) expression will directly affect single-cell V_mem_ states, given the dominant role of K^+^ transmembrane flux in V_mem_ regulation. It is unknown how these cell-level changes affect emergent pattern outcomes, therefore, we utilized simulation outcomes as a means of understanding the potential bioelectric landscape when the K^+^ supplementation conditions are reproduced in vitro. We expected to observe iso-potential bioelectric pattern signatures across cell cluster image regions, because high external K^+^ supplementation theoretically affects cell V_mem_ via direct changes to the potassium-specific Nernst (equilibrium) potential [46], and consequently, K^+^ V_mem_-dependent transmembrane ion flux, collectively bringing the cells closer to 0 mV. Our hiPSC single cell RNA-Sequencing (scRNA-Seq) data indicated that the voltage-gated K^+^ ion channels were more highly expressed than Na^+^ or Cl^−^ ion channels and supports the notion that they are likely the main contributor to voltage-gated ionic fluxes in the pluripotent state. Furthermore, Kv7.2 (KCNQ2) was the most highly expressed voltage-gated ion channel (Figures S3–S5). From this observation, we implemented a single voltage-gated potassium ion channel component in the model, which provided the dual effect of K^+^ control of single-cell voltage-dependent conductance and a consistent means to relate model cell parameter choice back to the experimentally measured cell current-voltage relationship from whole-cell patch clamp. Collectively, the simulation of K^+^ supplementation resulted in less observable differences from control conditions than the GJC perturbations (Figure 3) which are more challenging to discriminate by pattern classification techniques. 

**Figure 4:**
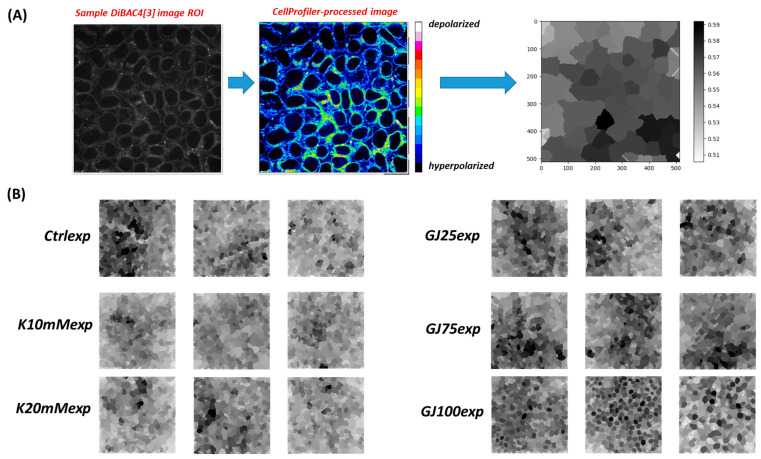
Experimental DiBAC image transformation pipeline for a sample image ROI. (**A**) The raw output image initially undergoes pre-processing upon export to ImageJ to generate a pseudo-colored representation of a DiBAC_4_(3) image containing local and global features of V_mem_ heterogeneity in an image ROI. Transformation of the CellProfiler image ROI contains a singular grayscale intensity for each cell, representing the averaged relative V_mem_ across the membrane boundary. (**B**) 3 sample output images from each representative in vitro culture condition-specific V_mem_ patterns highlight the complexity and inconspicuous differences in resultant bioelectric patterns. Refer to Table 1 for more details on conditions. Condition nomenclature: ‘Ctrlexp’ = Unmodified mTeSR culture media, experimentally-observed patterns; ‘GJ25exp’ = mTeSR culture media supplemented with 10 uM 18β-GA (gap junction inhibitor), experimentally-observed patterns; ‘GJ75exp’ = mTeSR culture media supplemented with 60 uM 18β-GA (gap junction inhibitor), experimentally-observed patterns; ‘K10mMexp’ = 10 mM KCl mTeSR culture media supplement, experimentally-observed patterns; ‘K20mMexp’ = 20 mM KCl mTeSR culture media supplement, experimentally-observed patterns; ‘GJ100exp’ = mTeSR culture media supplemented with Cx43 (GJA1) expression knocked down, experimentally-observed patterns.

Motivated by the model predictions, we imaged hundreds of human iPSC colonies under corresponding experimental conditions with manipulation of extracellular K^+^ or inhibiting/silencing of GJA1. The generation of bioelectric pattern images was facilitated by DiBAC_4_(3)-based experimental confocal microscopic assays [25]. DiBAC_4_(3) is an anionic fluorophore that partitions the cell membrane leaflets in accordance with cell membrane depolarization or hyperpolarization, thereby enabling steady-state qualitative observation of both cellular and multicellular V_mem_ patterns (see Section 2.3). DiBAC_4_(3) fluorescence intensity output images (Figure S12) were pre-processed to yield grayscale outputs for further analysis (Figure 4) (see Section 2.8 and Section 2.13 for detailed description). The ‘Ctrlexp’, ‘K10mMexp’, and ‘K20mMexp’ conditions shown on the left-hand side of Figure 4 clearly show minor differences in greyscale value (V_mem_ state) across the sample images. In contrast, the gap junction-altered culture conditions (Figure 4) by 18-β-GA or through CRISPRi of GJA1 result in isoelectric ‘salt-and-pepper’ V_mem_ domains, like those observed in [35] and predicted by the BETSE simulations output in Figure 3. While the trends across the two categories of perturbation conditions match our models qualitatively, we constructed a pipeline in order to quantitatively compare corresponding model and experiment conditions.

### 3.3. A Pipeline for Comparing Multicellular Bioelectric Patterns

**Figure 5:**
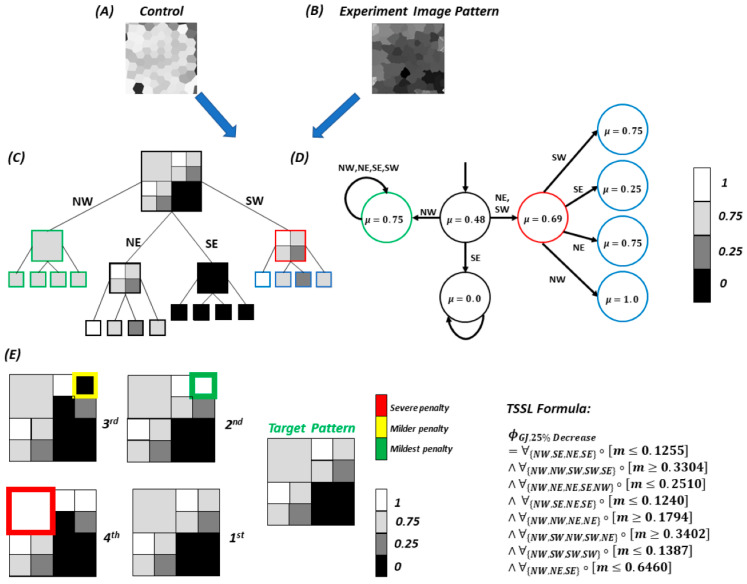
Simulated outcomes from BETSE (**A**) and experimental images (**B**) are compared through the development of pattern class formula and similarity scoring. The initial generated DiBAC_4_(3) microscopy image undergoes post-processing in ImageJ to obtain a grayscale representation of relative averaged V_mem_ for each cell; fluorescence intensity values are obtained for infinitesimal sections of each cell membrane and summated, then subsequently divided by cell circumference. This process standardizes pattern image outputs to a format similar to the simulations. The output images are initially transformed into quad-trees (**C**), data structures in which spatial regions of an image are sequentially subdivided into quadtrees until a particular region only contains a singular pixel intensity; value nodes and edges contain data specific to each image or image set. Each node contains the average grayscale intensity value and variance present in a particular spatial quadrant region and the edges contain information on the quadrant’s spatial region coordinates and level of resolution. (**D**) The quad transition system (QTS) accepts input quad-trees and generates a tuple containing information about grayscale intensity present in a specific spatial region and level of resolution. (**E**) Similarity scoring is performed in accordance with a metric known as ‘distance to satisfaction’; briefly each input image is compared against the TSSL formula describing a target pattern of interest. If the image violates a particular rule at a lower level of image resolution (i.e., whole image), a greater penalty (similarity score reduction) is given than if occurred at a higher level (zoomed in) of resolution, such as the red square versus the yellow square. Furthermore, the more different grayscale for a spatial region is from the target’s in that region, the greater the assessed penalty (yellow square versus the green square).

As evident from Figure 3 and Figure 4, the resting bioelectric patterns from non-excitable hiPSC colonies reflect a high degree of heterogeneity. Furthermore, while several trends are observed in bioelectrical properties upon perturbation with K^+^ addition or gap junction inhibition, a quantitative method is needed to quantify pattern similarity and quality of model output. Thus, the saved computational simulations and microscopy images were subsequently used for bioelectric pattern analysis and comparison in a supervised machine learning pipeline (see Section 2.14, Figure 5).

To obtain a pattern classifier for the simulated or experimental culture condition pattern profile, images comprising the simulated image set were denoted as the desired pattern profile (+), and the remaining culture condition-specific image sets were aggregated and collectively denoted as (−). The supervised machine learning algorithm (RIPPER) was then utilized to identify unique local and global features of the simulated condition image set. This process was repeated for each output simulated image set associated with the four altered culture conditions; for example, the ‘GJ25comp’ image class was denoted as the target (+) pattern profile and output images from the other four culture conditions were collectively designated (−) undesired pattern profiles. The classification algorithm performance is summarized in Table 1. Our simulated pattern classes yielded classification accuracies ranging between 72% and 98%, indicating that the image analysis pipeline could adequately discern differences in bioelectric pattern features amongst many of the simulated conditions.

**Figure 6:**
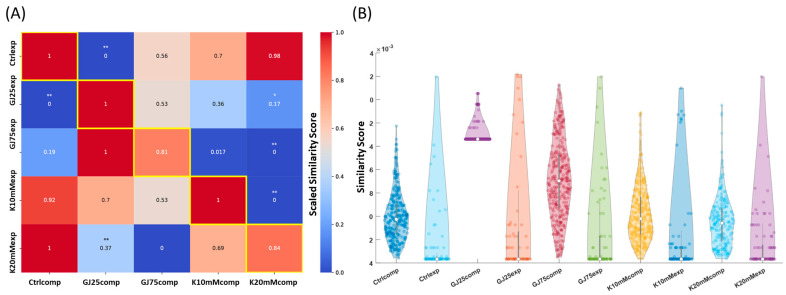
(**A**) Scaled similarity scoring outcomes are shown; each simulated culture condition (columns) is compared against in vitro patterns from the selected culture conditions (rows). Culture conditions analogous between the 2 domains (i.e., BETSE ‘control’ vs. in vitro ‘control’) are denoted by values on the matrix diagonal, surrounded by a yellow border. Statistically significant differences are indicated by double (*p* < 0.05) and single (*p* < 0.1) asterisks (located above score values). (**B**) The violin plot shows the similarity score distribution for each observed image in comparison to the simulated ‘GJ25comp’ target pattern profile. Condition nomenclature: ‘Ctrlcomp’ = Un-modified culture media, simulated patterns; ‘GJ25comp’ = Weak inhibition of gap junction cell-cell communication, simulated patterns; ‘GJ75comp’ = Strong inhibition of gap junction cell-cell communication, simulated patterns; ‘K10mMcomp’ = 10 mM KCl culture media supplement, simulated patterns; ‘K20mMcomp’ = 20 mM KCl culture media supplement, simulated patterns; ‘Ctrlexp’ = Unmodified mTeSR culture media, experimentally-observed patterns; ‘GJ25exp’ = mTeSR culture media supplemented with 10 uM 18β-GA (gap junction inhibitor), experimentally-observed patterns; ‘GJ75exp’ = mTeSR culture media supplemented with 60 uM 18β-GA (gap junction inhibitor), experimentally-observed patterns; ‘K10mMexp’ = 10 mM KCl mTeSR culture media supplement, experimentally-observed patterns; ‘K20mMexp’ = 20 mM KCl mTeSR culture media supplement, experimentally-observed patterns.

Previous use in multicellular pattern identification and parameter optimization efforts have validated the ability of this algorithm to distinguish complex image pattern features within biological multicellular systems [6,47]. Our observed classification accuracies were comparable to magnitudes recorded in previous [6,47] pattern identification efforts, which gave us confidence to utilize the pattern descriptors in downstream comparisons. We first sought to analyze similarity or dissimilarity amongst the five unique culture condition-based bioelectric pattern profiles. We hypothesized that the baseline heterogeneity present in the ‘Ctrlcomp’ profile would provide similar pattern profiles to those subjected to additional K^+^ media supplementation because connected cells can still communicate each other’s bioelectric state and maintain pattern heterogeneity. Conversely, we hypothesized that partial gap junction inhibition would produce dissimilar emergent bioelectric pattern profiles because the inability to communicate bioelectric state between connected cells would prevent them from maintaining native pattern heterogeneity amongst groups. To facilitate this pattern similarity assessment, the simulated ‘Ctrlcomp’ pattern classifier rule set was used as the desired pattern profile to be recreated in an input pattern image in the TSSL image analysis pipeline. Normalized similarity score outcomes are shown in the farthest left-hand column in Figure 6A; the ‘GJ25exp’ and ‘GJ75exp’ experiment image classes produced the lowest similarity scores relative to the images associated with the two K^+^ supplementation simulated culture conditions. The pattern similarity scoring outcomes and trends collectively suggests that gap junction communication is imperative to maintain local and global bioelectric pattern heterogeneity.

The image set associated with the fluorescent experimental recapitulation of the hiPSC computational model ‘Ctrlcomp’ simulation culture conditions generated the highest pattern similarity score when compared to the other experimentally produced image patterns (Figure 6A and Figure S1E). The ‘K10mMexp’ and ‘K20mMexp’ in vitro pattern classes produced similar simulation scores to the BETSE ‘Ctrlcomp’ pattern image set. The consistent trend of culture-condition associated pattern similarity holds across domains (Figure S1E), suggesting some unique spatial pattern relationship between these distinct condition pattern profiles. Further inspection of the pairwise relationships between experimental and computational conditions along the diagonal of the scoring matrix indicates the ability of the models to best capture patterns associated with the analogous colony microscopy images. This outcome indicates that complex bioelectric patterns can be accurately predicted, given adequate system component characterization. We observe a few caveats—for example the ‘K10mMcomp’ pattern class possessed the highest observed quantitative similarity score when compared to its’ experimental ‘K10mMexp’ analog but did not produce statistically significant differences in similarity score from other culture condition-specific pattern classes (Figure 6A). Furthermore, the ‘Ctrlexp’ condition outperforms the K^+^ experiment classes in both ‘K10mMexp’ and ‘K20mMexp’ cases comparatively, which indicates that K^+^ supplementation may not significantly vary local or global steady-state bioelectric patterns.

Next, we evaluated the hypothesized effect of the partial gap junction block by comparing discernable pattern profile differences between conditions by comparing each of the modeling and experimental image sets to the ‘GJ25comp’ pattern class (Figure 6B). In this analysis (corresponding to the second column of the matrix in Figure 6A as well as all other simulated pattern classes), the β-GA-associated pattern classes produced higher pattern similarity scores than the K^+^ supplemented conditions pattern similarity scores. This ordering of pattern similarity aligns with our expectations, as K^+^ supplementation should not necessarily alter large-scale (multicellular) V_mem_ heterogeneity while β-GA, the gap junction uncoupling agent, increases the likelihood of iso-potential cell cluster formation throughout colonies. We further assessed pattern similarity between the ‘GJ25comp’ and ‘GJ75comp’ classes and the corresponding analog experimental culture condition-associated classes, ‘GJ25exp’ and ‘GJ75exp’. From the matrix of scoring outcomes (Figure 6A), we note that the ‘GJ25exp’ culture condition patterns produced the highest similarity score in comparison to the other experimental perturbation classes, and the next highest perturbation class similarity score derived from the ‘GJ75exp’ class (Figure 6A). This quantitative similarity trend supported the hypothesized multicellular pattern effect in GJ trans-junctional current decrease proposed by [35,41]; the ‘GJ25comp’ class showed statistically significant differences in similarity scores with respect to the ‘Ctrlexp’ and ‘K20mMexp’ in vitro pattern classes (Figure 6B and Figure S8). The ‘GJ75comp’ class did not display statistically significant differences in average similarity scores with respect to ‘GJ25comp’ class, but the highest similarity score was observed to come from the analog ‘GJ75exp’ culture condition pattern class and the precision-recall curve for the ‘GJ75comp’ simulated classifier outperformed all other pattern classes (Figure S13). Correspondingly, the ‘GJ75comp’ image set contained the highest average similarity scores. Furthermore, this image set contained the least amount of variance in scores, as seen from the distribution in (Figure S8). Among the simulated classes the ‘GJ75comp’ condition possessed the next highest average pattern similarity scores, and most of these scores aggregate near the distribution mean (Figure S1A). This representation of pattern similarity provides a clearer picture of the machine learning algorithm’s ability to distinguish target pattern profiles and pattern profiles with similar defining spatial features. These same trends in score heterogeneity is observed in the ‘GJ75comp’ target profile violin and bar plot (Figures S1B and S8).

The two β-GA pattern class similarity scores show clear distinction over the K^+^ supplementation classes, consistent with trends observed earlier concerning the relative pattern similarity between control and K^+^ supplemented culture pattern outcomes. Although the highest average image set pattern similarity scores were observed for the ‘K10mMexp’ and ‘K20mMexp’ in vitro culture condition image pattern sets, many of the highest scoring individual images were associated with the ‘GJ100exp’ and ‘GJ75exp’ culture conditions (Figure S8). While these results did not match our expectations that the highest scores would match the simulated ‘GJ75comp’ class or in vitro ‘GJ75exp’ class, they do suggest that experimental pattern profiles associated with attenuated gap junction communication are likely similar to the salt-and-pepper type ‘isolated’ iso-electric cell clusters displayed by the simulation. Overall, the model was able to distinguish unique local and global spatial features of bioelectric patterning that arise from partial inhibition of cell-cell communication. Finally, despite the observed visual similarity between the BETSE simulated ‘GJ95comp’ condition and the ‘GJ100exp’ condition images, the 10-fold cross-validation performed on the machine learning training data revealed poor correct classification rate (0.47) for the ‘GJ25comp’ target pattern profile (Table A1). The poor performance was attributed to the unique global dark grey characteristic of image pattern profiles, as opposed to the sample simulated images shown for ‘Ctrlcomp’, ‘GJ25comp’, ‘GJ75comp’, ‘K10mMcomp’, and ‘K20mMcomp’ (Figure 3). Collectively, the similarity scores indicated that the model is capable of predicting tissue-level features that are analogous to experimental bioelectric patterns.

### 3.4. Particle Swarm Optimization Highlights Parameters That Potentially Lead to Desired Bioelectric Patterns

We extended the analysis of the computational model performance to probe the model’s capacity to automatically generate conditions that would lead the cells to self-organize into desired bioelectric pattern profiles. Since the TSSL pipeline assigns quantitative values to images based on similarity to a target image, we implemented Particle Swarm Optimization (PSO) [48] to maximize and thus improve the similarity score between input image sets and the target pattern classifier through selection of more optimal parameter assignments. The algorithm initializes simulated parameters, or particles, at maximum and minimum extremes of the allowable n-dimensional parameter space, then iteratively searches for local maxima from the similarity score space and compares maximum scores amongst local swarms, and the maximum or minimum-valued scores from each group are assumed to be the optimized parameter(s). These optimized parameters are interpreted as the needed parameter conditions to ensure a desired target image patterning outcome. The algorithm is described in further detail in Appendix A.

Based upon our measurements of single cell heterogeneity in ion channel expression, we initially set up an optimization strategy in which individual ionic transmembrane ‘diffusivities’ could vary freely in a defined parameter space. We ran a two-parameter PSO search in which D_mem,K^+^_ and D_mem,Na^+^_ were simultaneously varied throughout the allowable parameter spaces and subsequently simulated. This parameter space was defined between 1 × 10^−16^ and 1 × 10^−19^ m^2^/s for K^+^ diffusivities and 1 × 10^−17^ to 1 × 10^−19^ for both Na^+^ and Cl^−^ diffusivities; the fractional gap junction surface area parameter ($\beta_{GJ}^{o}$) was set to cover space between 1 × 10^−9^ (completely closed) and 1 × 10^−7^ (100% open) values. Importantly, the characteristic steady-state D_mem,K^+^_:D_mem,Na^+^_ ratio deserves more attention than the individual D_mem,K^+^_ and D_mem,Na^+^_ values, due to the downstream consequences on cellular V_mem_ state. The Goldman Hodgkin Katz equation shows that when intra- and extracellular ion concentrations are kept constant, the ionic diffusivity ratio directly controls V_mem_ state, with this trend being validated experimentally [35,49,50,51,52]. Therefore, we assumed that the preservation of ionic permeability ratio is the primary determining factor for control steady-state V_mem_ and surmised that novel D_mem,K^+^_:D_mem,Na^+^_ ratio values would relatively recapitulate that of the original ‘Ctrlcomp’ pattern class if applied in vitro. Each parameter-specific ‘particle’ moved about in the allowable parameter search-space’ in accordance with both its personal and group best similarity score each iteration until either a defined error threshold was met, or the produced pattern similarity score in comparison to the target pattern is maximized for the current input parameter(s). The resulting PSO search arrived at a D_mem,K^+^_:D_mem,Na^+^_ ratio value of 10 from the starting assignment of 6.13.

We repeated this procedure for the ‘GJ25comp’ and ‘GJ75comp’ target pattern profiles. The relative adherence of the predicted K^+^:Na^+^ diffusivity ratios to that of the original simulated ‘Ctrlcomp’ class value gave us confidence that in vitro recapitulation of these values would produce patterns of a similar nature to that of the unaltered mTeSR ‘Ctrlcomp’ culture condition. This led us to inquire into the quantitative pattern similarity between the novel simulated image set patterns classifiers and its’ ‘Ctrlexp’ in vitro culture condition analog. We next extended this novel parameter optimization analysis to in vitro pattern similarity comparison.

**Figure 7:**
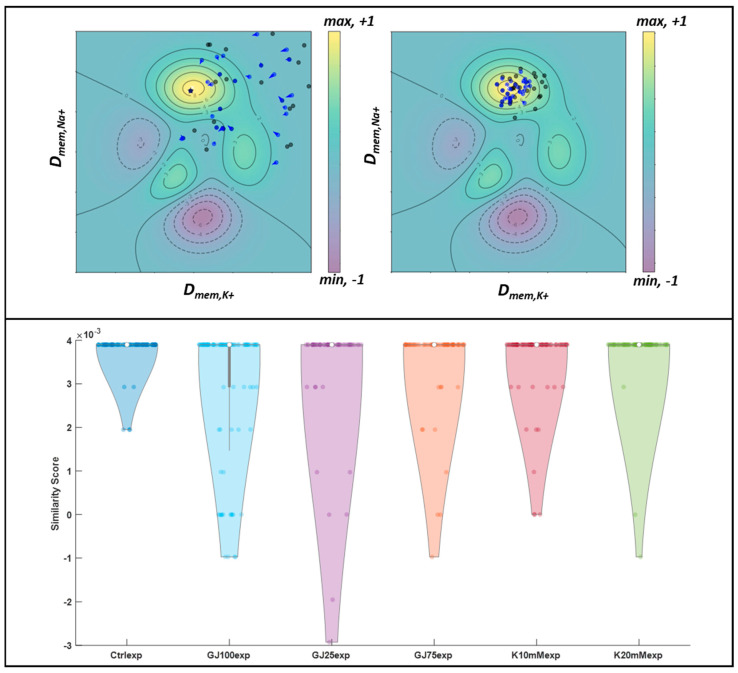
A schematic layout for parameter optimization (**top panel**) Simulations containing differing parameter combinations are run and subsequently scored for similarity to the desired target pattern; if the similarity score increases as a particle searches a certain direction it will continue to move until the maximum target similarity is obtained, at which point the algorithm stores the parameters and defines them as optimal parameters to produce a desired bioelectric pattern. (**Bottom**): Similarity scoring between optimized parameter associated ‘Ctrlcomp’ simulation pattern classifiers and experimental condition pattern sets. Condition nomenclature: ‘Ctrlexp’ = Unmodified mTeSR culture media, experimentally-observed patterns; ‘GJ25exp’ = mTeSR culture media supplemented with 10 uM 18β−GA (gap junction inhibitor), experimentally-observed patterns; ‘GJ75exp’ = mTeSR culture media supplemented with 60 uM 18β−GA (gap junction inhibitor), experimentally-observed patterns; ‘K10mMexp’ = 10 mM KCl mTeSR culture media supplement, experimentally-observed patterns; ‘K20mMexp’ = 20 mM KCl mTeSR culture media supplement, experimentally-observed patterns; ‘GJ100exp’ = mTeSR culture media supplemented with Cx43 (GJA1) expression knocked down, experimentally-observed patterns.

We hypothesized that the optimized D_mem,K^+^_/D_mem,Na^+^_ parameter sets predicted to optimally satisfy ‘Ctrlcomp’ target pattern conditions would reproduce similarity score relationships when compared to in vitro pattern classes, analogous to the manner that the original BETSE ‘Ctrlcomp’ pattern class was compared against in vitro pattern classes. To this end, we utilized the optimal ion diffusivity parameters produced from the PSO combined D_mem,K^+^_/D_mem,Na^+^_ as input conditions to iterative BETSE simulations (n ≥ 200), and we subsequently assigned novel pattern classifiers to both image sets (Figure 7, bottom). These novel ‘Control PSO’ image pattern classifiers were defined as the target pattern profile with respect to the image sets pertaining to in vitro recreation of the simulated culture conditions. Interestingly, similarity scores from the in vitro ‘Ctrlexp’ conditions performed the best with respect to both K^+^ supplementation and gap junction inhibition culture conditions (Figure 7). This result was to be expected, as the automated parameters defined by the model were trained to replicate pattern features of the resting culture condition. Although this similarity score differences were not statistically significant, the same trends were observed in Figure 6 with respect the degree of similarity of the perturbations to the target control, suggesting an order of conserved hierarchy in image pattern similarity trends across recapitulated culture conditions. 

### 3.5. Disruption of Intrinsic Gap Junction Connectivity in iPSC Colonies Leads to Novel Pattern Formation

**Figure 8:**
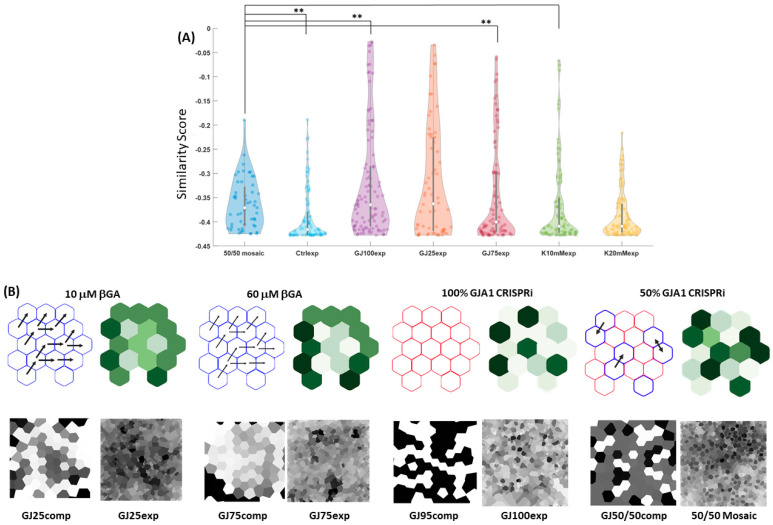
(**A**) The similarity score violin plot is shown for the pattern comparison between condition-specific experiment image sets and the target ‘50/50 mosaic’ pattern classifier. Statistically significant differences between the ‘50/50 mosaic’ similarity score and the other pattern classes are indicated by double (*p* < 0.05) asterisks (located above the violin plot). (**B**) Schematics of the various modulations to gap junction communication and resultant multicellular patterning of V_mem_ from pharmacological or genetic approaches. Arrows indicate directionality and magnitude of ion flow based upon Vmem. Below each condition are example BESTE simulation (**left**) and processed hiPSC microscopy image (**right**) of colonies. Condition nomenclature: ‘Ctrlexp’ = Unmodified mTeSR culture media, experimentally-observed patterns; ‘GJ25exp’ = mTeSR culture media supplemented with 10 uM 18β-GA (gap junction inhibitor), experimentally-observed patterns; ‘GJ75exp’ = mTeSR culture media supplemented with 60 uM 18β-GA (gap junction inhibitor), experimentally-observed patterns; ‘K10mMexp’ = 10 mM KCl mTeSR culture media supplement, experimentally-observed patterns; ‘K20mMexp’ = 20 mM KCl mTeSR culture media supplement, experimentally-observed patterns; ‘GJ100exp’ = mTeSR culture media supplemented with Cx43 (GJA1) expression knocked down in all cells, experimentally-observed patterns; ‘GJ95comp’ = simulated GJA1 CRISPRi colony patterns assuming 5% isoform compensation;; ‘GJ50/50comp = simulated patterns for the mosaic condition; ‘50/50 mosaic’ = 50% wild-type WTC11 hiPSCs, 50% Cx43 (GJA1) CRISPR-knockdown WTC11 hiPSCs mixed, cultured in mTeSR culture media, experimentally-observed patterns.

The hiPSC bioelectric model reproduces experimental patterning outcomes, whether by perturbing intrinsic cellular properties with small molecular inhibitors or altering external culture conditions; however, confidence in model capability can be further tested by prediction of multicellular pattern profiles when unfitted, new conditions are tested experimentally. The hiPSC-specific bioelectric model simulations and in vitro ‘GJ25exp’ and ‘GJ75exp’ results suggested that attenuation of gap junction communication between neighboring hiPSCs would yield novel V_mem_ patterns at the tissue scale. To this end, we simulated a novel co-culture condition, which we named ‘GJ 50/50 comp’, in which simulated cells were defined by two different resting V_mem_ states and gap junction activities; one population contained normal GJA1-based gap junction dynamics, while the other implemented a complete block of gap junction cell-cell conductance mimicking silencing expression of the connexin isoform. Following the creation of a simulation image set, we utilized TSSL to define a pattern classifier for the simulated mosaic condition (n = 200) (Figure 8). This effect could not be replicated via small molecule β-GA use, as it only provides partial conductance inhibition between adjacent hiPSCs. Therefore, we cultured two hiPSC lines (LBC2 and LBC2-GJA1) to replicate these simulated mosaic conditions in vitro. LBC2-GJA1 is the CRISPRi-edited version of the LBC2 hiPSC line in which GJA1 (Cx43) is silenced with continuous doxycycline exposure. We cultured the cells for four passages before experimental characterization and seeded the corresponding cell populations at approximate equal densities for 1 day prior to imaging; the resultant 50/50 mosaic phenotypic mix was imaged using DiBAC. 

We specifically compared this ‘50/50 mosaic’ condition to the other in vitro perturbation and control image sets. Ideally, full silencing of this specific isoform would ablate all intercellular ion flux, but multiple studies have shown the existence of other expressed connexin isoforms, which can form homotypic or heterotypic connexon intercellular ion channels [53] and result in residual coupling of intercellular ion fluxes. The novel pattern class addition allows comparison of the quantitative pattern similarity between a “complete” gap junction communication inhibition and a mosaic condition of GJA1 silenced and wild-type cells. We performed similarity score analysis for the collective in vitro classes with respect to the target novel experimental ‘50/50 mosaic’ pattern classifier and found that this specific bioelectric pattern niche possessed the highest similarity score in comparison to the simulated analog ‘GJ50/50comp’ target pattern classifier. This measured pattern similarity outcome supported the hypothesized effect on emergent multicellular bioelectric patterning, even i when multiple parameters of the bioelectric system are simultaneously perturbed. Interestingly, there were statistically differences in similarity score between the simulated ‘GJ50/50comp’ pattern set and the corresponding ‘50/50 mosaic’ experiment condition and both ‘GJ25exp’ and ‘GJ75exp’ gap junction inhibition experiment pattern classes, as well as the ‘K20mMexp’ pattern class. The ‘50/50 mosaic’ class did not produce the highest average similarity score (Figure 8A) with respect to the other explored in vitro conditions; however, the highest scoring median values came from the ‘50/50 mosaic’ and ‘GJ25exp’ culture condition image sets, and many of image scores are distributed evenly across the range of covered values; other conditions, such as ‘Ctrlexp’ and ‘GJ25exp’, are heavily concentrated towards the bottom of their respective score ranges. These score trends, coupled with some high performing outlier images, could explain the highest mean scores belonging to the unexpected ‘GJ25exp’ condition. Regardless, the highest performing scores belonging to this class are interpretable when β-GA pattern effects are considered. The compound blocks intercellular gap junction communication; consequently, local cells or clusters containing ‘isolated’ V_mem_ states cells would be an expected pattern profile feature that is partially replicated in the ‘50/50 mosaic’ culture condition. β-GA media addition inhibits cell-cell ion flux across the whole cell population, so the same expected locally distinct V_mem_ heterogeneity amongst neighboring cells should be present in this co-culture. We observed a significant difference in quantitative pattern similarity when a portion of the colony cells have intercellular communication inhibited. Specifically, the ‘GJ25exp’, ‘GJ100exp’, ‘Ctrlexp’, and ‘K20mMexp’ image sets produced statistically significant mean similarity scores with respect to the ‘50/50 mosaic’ class. These distinct score differences suggest that the pattern consequences resulting from these slight alterations in the bioelectric system conditions are recapitulated even when compared to similar culture conditions. However, the difference between the ‘50/50 mosaic’ condition and ‘K10mMexp’ and ‘GJ75exp’ experimental patterns were not statistically significant. Overall, these observed trends provide insight into the specific local and global pattern characteristics that are similar between the gap junction and K^+^ supplementation perturbation patterns, as the relatively high similarity scores amongst the ‘Ctrlexp’ and ‘K20mMexp’ pattern culture conditions lend credence to the basal V_mem_ spatial heterogeneity that is present in these specific culture conditions. 

## 4. Discussion

Autonomous control of cell patterning during embryogenesis or morphogenesis integrates multiple intrinsic and extrinsic dynamic factors to achieve repeatable target morphological outcomes, such as the consistent emergent pattern classes that we report in this study. Understanding the underlying molecular components influencing these outcomes could open the door for leveraging the bioelectrical control mechanism of morphogenic trajectories. Previous studies have highlighted the role of ion channels defects in establishing altered anatomical trajectories in multiple species [12]; however, few to date [14] have computationally addressed the consequences of channelopathies across multiple scales (molecular, cellular, and multicellular). Interestingly, studies have shown Kir2.1 to be directly implicated in the onset of craniofacial defects in humans [54] and this effect has been recapitulated in frog and Drosophila embryos [55]. The conserved role in the emergence of downstream anatomical defects across species lends credence to a potential canonically conserved V_mem_-current relationship for the inwardly-rectifying potassium channel. Thus, other human ion channel homologs may be probed for their role in developmental morphological trajectory establishment and maintenance via computational simulations. Therefore, we implemented a novel bioelectric systems characterization of hiPSC bioelectric pattern dynamics to predict emergent outcomes under normal (‘Control’) and altered cell culture conditions, specifically designed to probe the role of lower-level components in pattern instruction. This characterization design serves the dual purpose of understanding how individual bioelectric components work in an integrated manner to create stable bioelectric pattern outcomes and how specific perturbations to the individual or combined system components lead to predictable steady-state patterning outcomes. We observed that our 5 simulated novel culture conditions and resulting bioelectric patterns were quantitatively similar on a global and local scale. The similarity scores values observed are comparable to those observed in [6]; furthermore, the variability present in experimental pattern profile score distributions in [6] was also observed in this study. This multi-level integration effort demonstrated the capacity to predict novel pattern features under defined certain cell culture conditions. We extended the pattern prediction effort to an unexplored culture niche in which two populations of hiPSCs containing unique cell bioelectric characteristics are co-cultured in a mosaic manner. Simulated patterns replicating the co-culture conditions were quantitatively similar to that of the experimentally replicated ‘50/50 mosaic’ co-culture condition. Importantly, this validates the model’s predictive utility under both well-defined and partially defined initial bioelectric system conditions. Ultimately, this model-guided pattern prediction represents a critical tool that enables the prediction and understanding of emergent bioelectric system dynamics in vitro.

While our approach shows promise for simulating new bioelectric conditions that drive tissue patterning, the model’s utility in translational applications is hindered by several factors. First, the model presented in this work is a simplified representation of single-cell bioelectric components and their activities; we assumed Na^+^ and Cl^−^ to lack V_mem_-specific ion channel dynamics and assumed a single ion channel K^+^ characterization; this does not occur realistically, as even a homogenous pluripotent stem cell population possess a distribution of voltage-gated K^+^ ion channel expression and respective channel activity. This model drawback could be potentially alleviated by the integration of transcriptomic data and more specifically designed patch clamp assays. Furthermore, we populated the model to possess Cx43 (GJA1) V_mem_-dependent gap junction conductance. In reality, hiPSCs possess a distribution of gap junction expression and isoform types, each isoform possessing slightly different V_mem_ sensitivities. This lack of phenomenological model representation naturally causes model simulations to lose predictive accuracy even if all other relevant intrinsic and extrinsic variables and parameters are properly accounted for. Also, the model does not account for changes to ion channel and gap junction activity dynamics after the simulation begins. These system components are subject to post-translational modifications and epigenetic modifications, as evidenced by the negative effect of nicotine (a teratogen) on HCN2 function during *X. laevis* neural crest development [14]. This consequently means that even an hiPSC model that fully incorporates transcriptomic and proteomic data could fail to produce solutions that reflect in vitro patterning outcomes if the functional consequences of these modifications are not incorporated into model channel function characteristic equations. These potential effects are simplified to avoid overfitting and unnecessarily over-complicating model structure in efforts to preserve predictive flexibility and to guide insights for specific model improvements [56]. Once properly trained and tuned with more experimental datasets and better biotechnological tools, such as ArcLight [57], model bioelectric systems may be probed and assessed for their temporal evolution properties and how they are affected by timed changes to aspects of the bioelectric machinery or culture environment. One promising tool that already offers a high level of temporal precision in its signal control is optogenetic ion channels, which have already shown applicability in mouse embryonic stem cells [58].

The novelty of this study extends past the validated multicellular biophysics; this bridge between the computational and in vitro domain space makes model-based bioelectric pattern prediction and control realistically applicable to artificial living machine design from iPSCs [3]. Gap junctions not only possess dynamics pertaining to the ‘like’ cell population but may also isolate differing cell phenotypes or sub-populations [59,60] in larger multicellular systems, suggesting pivotal bioelectric pre-patterning roles in development. Because we observed that pharmacological inhibition of GJCs (β-GA media addition) led to unique spatial bioelectric pattern features, we hypothesized that a heterogeneous mixture of GJC disruption would yield a novel class of bioelectrical patterns. The defining feature of this novel pattern configuration is the way the bioelectric system components are altered. Rather than altering the system component’s characteristics throughout the entire cell population, the dynamics would be altered for only a certain cell subpopulation within the collective. The ability to accurately predict pattern outcomes without prior assay-derived component dynamics probing would validate the model’s ability to not only predict multicellular pattern outcomes under well-defined system component modifications but, would also inspire confidence to further predict and analyze previously undefined bioelectric pattern conditions in vitro. Importantly, the experimentally testable spectrum of culture conditions and potential combined system parameter modifications would exponentially decrease both time and required resources in laboratory settings. Furthermore, the particle swarm optimization results indicate that cell dynamics may function in different regions of the cell bioelectric parameter space and still create similar and robust patterning outcomes; thus, simulation-guided ionophore or gap junction supplement culture design can be used to test the feasibility of engineering artificially induced bioelectric properties to obtain congruent end patterns in vitro. Synthetic control over form would extend potential use for bioelectric patterning in various practical applications. For example, transmembrane ion diffusivities are bioelectric system parameters that can be pharmacologically tuned in vitro with ionophore supplementation. Ionophores are compounds that enhance ion transport across the cell membrane via a pore-forming channel mechanism; different classes of ionophores selectively transport specific ions, enabling control over individual transmembrane ionic fluxes. Furthermore, integration of microelectrode arrays (MEAs) into cell cultures may also enable precise spatial control over ionophore concentration [61,62], which would afford a higher degree of precision spatial ion flux regulation. Importantly, the ability to correctly predict bioelectric system parameter combinations in physiologically reasonable search space would enable precise planning of ionophore and culture media ionic composition to controllably manipulate bioelectric patterns through intrinsic/autonomous means. 

## 5. Conclusions

Overall, outcomes from these various parameter optimizations demonstrate the potential applications to multicellular system bioelectric pattern control and highlight that further model improvements must first be made before reliable application to hiPSC cellular systems. The role of gap junctions in bioelectric intercellular communication is conserved across species in the context of cell fate control and has been shown to disrupt spatial differentiation trajectories and regulate information and cell state processing propagation from single-cell to multicellular scale [5,18]. Model-guided regulation of gap junction dynamics may be utilized in efforts to identify unique, novel multicellular pattern types and potential control over positional outcomes during morphogenesis or tissue regeneration. Ultimately, the novel bottom-up bioelectric system characterization enabled the ability to predict and control bioelectric patterning in homogenous hiPSC cell clusters in vitro, opening the possibility to engineer control over bioelectric pattern design and subsequently ensure robust spatiotemporal morphogenic trajectories. 

## Figures and Tables

**Table 1:** Image set size data and performance statistics for the machine learning classification algorithm.

Simulated Condition	# of Images	Correct Pattern ID % (Training Set)	F-Measure	Precision	Recall	Experimental Condition	# of Images	Normalized Similarity Score
Control (Ctrlcomp)	282	90.63%	0.686	0.836	0.645	Control (Ctrlexp)	89	1
25% GJC inhibition (GJ25comp)	393	75.2%	0.681	0.672	0.726	βGA 10 μM (GJ25exp)	95	1
75% GJC inhibition (GJ75comp)	298	98.9%	0.969	0.969	0.968	βGA 60 μM (GJ75exp)	85	0.81
K^+^ 10 mM (K10mMcomp)	377	72%	0.688	0.678	0.737	K^+^ 10 mM (K10mMexp)	86	1
K^+^ 20 mM (K20mMcomp)	199	89.7%	0.828	0.818	0.842	K^+^ 20 mM (K20mMexp)	93	0.84
95% GJC block (GJ95comp)	199	99.5%	0.929	0.951	0.922	100% Cx43 KD, GJA1 CRISPRi cells (GJ100exp)	90	0.47

## Data Availability

All custom code and images analyzed in the paper can be accessed on Github: https://github.com/andre-norfleet/BETSE-repo (accessed on 1 December 2022).

## Supplementary Materials

The following supporting information can be downloaded at: https://www.mdpi.com/article/10.3390/cells13131136/s1, Supplementary text: TSSL syntax describing each pattern classifier; Figure S1: Similarity scores are shown for comparisons between BETSE culture condition target pattern classifiers. (A) Simulated ‘GJ25comp’ classifier quantitative comparison to other BETSE and in vitro condition-specific image patterns. (B) Simulated ‘GJ25comp’ classifier quantitative comparison to other BETSE and in vitro condition-specific image patterns. (C) Simulated ‘K10mMcomp’ classifier quantitative comparison to other BETSE and in vitro condition-specific image patterns. (D) Simulated ‘K20mMcomp’ classifier quantitative comparison to other BETSE and in vitro condition-specific image patterns. (E) Simulated ’Ctrlcomp‘ classifier quantitative comparison to other BETSE and in vitro condition-specific image patterns. Condition nomenclature: ‘Ctrlcomp’ = Un-modified culture media, simulated patterns; ‘GJ25comp’ = Weak inhibition of gap junction cell-cell communication, simulated patterns; ‘GJ75comp’ = Strong inhibition of gap junction cell-cell communication, simulated patterns; ‘K10mMcomp’ = 10 mM KCl culture media supplement, simulated patterns; ‘K20mMcomp’ = 20 mM KCl culture media supplement, simulated patterns.; Figure S2: Expression of Connexin 43 in hiPSC clusters, as indicated by Cx43 (GJA1) immunocytochemical staining. Left: WTC11 clusters, Cx43 (GJA1) is shown in green and the nuclear Hoechst33342 stain is shown in blue. Middle: LBC2 hiPSC clusters (non-silenced), Cx43 (GJA1) is shown in pink and the nuclear Hoechst33342 stain is shown in blue. Right: LBC2-GJA1 hiPSC clusters upon stable doxycycline-induced gene knockout, Cx43 (GJA1) is shown in pink and the nuclear Hoechst33342 stain is shown in blue.; Figure S3: Single-cell RNA sequencing expression profiles are shown for: voltage-gated K^+^ ion channel isoforms Kv7.2 (KCNQ2), Kv9.3 (KCNS3), Kv11.1 (KCNH2), Kv2.1 (KCNB1), Kv4.2 (KCND2), Kv3.4 (KCNC4), and Kir2,1 (KCNJ2); voltage-gated Na^+^ ion channel isoforms Nav1.6 (SCN8A), and Nav1.9 (SCN11A); Na^+^/K^+^-ATPase *α* (ATP1A1) and *β* (ATP1B1) subunit isoforms, and GAPDH in WTC11 hiPSCs; Figure S4: Single-cell RNA sequencing expression profiles are shown for: Cx40 (GJA5), Cx43 (GJA1), Cx36 (GJD2), and Cx45 (GJC1) connexin isoforms in WTC11 hiPSCs; Figure S5: Single-cell RNA sequencing expression profiles are shown for: voltage-gated Na^+^ ion channel isoforms Nav1.6 (SCN8A), and Nav1.9 (SCN11A) in WTC11 hiPSCs; Figure S6: Whole-cell patch clamp data is shown for (A) individual samples (n=9) are patched at their respective resting membrane voltage potentials (V_mem_); (B) transmembrane current density in response to increasing steady-state electrode holding potentials (mV); the dark line represents linear fit (R^2^ = 0.9729) between the holding voltages and whole-cell transmembrane current density; the derived curve was used for downstream ionic flux parameterization.; Figure S7: Violin plot of the similarity score distribution for each image set in comparison to the simulated ‘Ctrlcomp’ target pattern profile. Condition nomenclature: ‘Ctrlcomp’ = Un-modified culture media, simulated patterns; ‘GJ25comp’ = Weak inhibition of gap junction cell-cell communication, simulated patterns; ‘GJ75comp’ = Strong inhibition of gap junction cell-cell communication, simulated patterns; ‘K10mMcomp’ = 10 mM KCl culture media supplement, simulated patterns; ‘K20mMcomp’ = 20 mM KCl culture media supplement, simulated patterns; ‘Ctrlexp’ = Un-modified mTeSR culture media, experimentally-observed patterns; ‘GJ25exp’ = mTeSR culture media supplemented with 10 μM 18β-GA (gap junction inhibitor), experimentally-observed patterns; ‘GJ75exp’ = mTeSR culture media supplemented with 60 μM 18β-GA (gap junction inhibitor), experimentally-observed patterns; ‘K10mMexp’ = 10 mM KCl mTeSR culture media supplement, experimentally-observed patterns; ‘K20mMexp’ = 20 mM KCl mTeSR culture media supplement, experimentally-observed patterns; Figure S8: Violin plot of the similarity score distribution for each image set in comparison to the simulated ‘GJ75comp’ target pattern profile. Condition nomenclature: ‘Ctrlcomp’ = Un-modified culture media, simulated patterns; ‘GJ25comp’ = Weak inhibition of gap junction cell-cell communication, simulated patterns; ‘GJ75comp’ = Strong inhibition of gap junction cell-cell communication, simulated patterns; ‘K10mMcomp’ = 10 mM KCl culture media supplement, simulated patterns; ‘K20mMcomp’ = 20 mM KCl culture media supplement, simulated patterns; ‘Ctrlexp’ = Un-modified mTeSR culture media, experimentally-observed patterns; ‘GJ25exp’ = mTeSR culture media supplemented with 10 μM 18β-GA (gap junction inhibitor), experimentally-observed patterns; ‘GJ75exp’ = mTeSR culture media supplemented with 60 μM 18β-GA (gap junction inhibitor), experimentally-observed patterns; ‘K10mMexp’ = 10 mM KCl mTeSR culture media supplement, experimentally-observed patterns; ‘K20mMexp’ = 20 mM KCl mTeSR culture media supplement, experimentally-observed patterns.; Figure S9: Violin plot of the similarity score distribution for each image set in comparison to the simulated ‘K10mMcomp’ target pattern profile. Condition nomenclature: ‘Ctrlcomp’ = Un-modified culture media, simulated patterns; ‘GJ25comp’ = Weak inhibition of gap junction cell-cell communication, simulated patterns; ‘GJ75comp’ = Strong inhibition of gap junction cell-cell communication, simulated patterns; ‘K10mMcomp’ = 10 mM KCl culture media supplement, simulated patterns; ‘K20mMcomp’ = 20 mM KCl culture media supplement, simulated patterns; ‘Ctrlexp’ = Un-modified mTeSR culture media, experimentally-observed patterns; ‘GJ25exp’ = mTeSR culture media supplemented with 10 μM 18β-GA (gap junction inhibitor), experimentally-observed patterns; ‘GJ75exp’ = mTeSR culture media supplemented with 60 μM 18β-GA (gap junction inhibitor), experimentally-observed patterns; ‘K10mMexp’ = 10 mM KCl mTeSR culture media supplement, experimentally-observed patterns; ‘K20mMexp’ = 20 mM KCl mTeSR culture media supplement, experimentally-observed patterns.; Figure S10: Violin plot of the similarity score distribution for each image set in comparison to the simulated ‘K20mMcomp’ target pattern profile. Condition nomenclature: ‘Ctrlcomp’ = Un-modified culture media, simulated patterns; ‘GJ25comp’ = Weak inhibition of gap junction cell-cell communication, simulated patterns; ‘GJ75comp’ = Strong inhibition of gap junction cell-cell communication, simulated patterns; ‘K10mMcomp’ = 10 mM KCl culture media supplement, simulated patterns; ‘K20mMcomp’ = 20 mM KCl culture media supplement, simulated patterns; ‘Ctrlexp’ = Un-modified mTeSR culture media, experimentally-observed patterns; ‘GJ25exp’ = mTeSR culture media supplemented with 10 μM 18β-GA (gap junction inhibitor), experimentally-observed patterns; ‘GJ75exp’ = mTeSR culture media supplemented with 60 μM 18β-GA (gap junction inhibitor), experimentally-observed patterns; ‘K10mMexp’ = 10 mM KCl mTeSR culture media supplement, experimentally-observed patterns; ‘K20mMexp’ = 20 mM KCl mTeSR culture media supplement, experimentally-observed patterns.; Figure S11: Violin plot of the similarity score distribution for each observed image set in comparison to the simulated ‘GJ100comp’ target pattern profile. Condition nomenclature: ‘Ctrlexp’ = Un-modified mTeSR culture media, experimentally-observed patterns; ‘GJ25exp’ = mTeSR culture media supplemented with 10 μM 18β-GA (gap junction inhibitor), experimentally-observed patterns; ‘GJ75exp’ = mTeSR culture media supplemented with 60 μM 18β-GA (gap junction inhibitor), experimentally-observed patterns; ‘GJ100exp’ = mTeSR culture media supplemented with Cx43 (GJA1) expression knocked down, experimentally-observed patterns; ‘K10mMexp’ = 10 mM KCl mTeSR culture media supplement, experimentally-observed patterns; ‘K20mMexp’ = 20 mM KCl mTeSR culture media supplement, experimentally-observed patterns; Figure S12. The raw output experiment DiBAC_4_(3) images of the 60 μM addition of β-GA culture condition; Figure S13. The precision-recall curves are shown for the five pattern classifiers defining the simulated culture conditions.

## Appendix A

### Appendix A.1. Estimated Single-Ion Flux Contribution

The relative contribution of $I_{K}$, $I_{Na}$, $I_{GJ}$, and $I_{pump}$ at a V_mem_ was estimated via linear regression; specifically, K^+^-ion flux was assumed to be the difference between measured transmembrane ionic current density and simulated collective passive Na^+^ and Cl^−^ ion fluxes, Na^+^/K^+^-ATPase associated active ion fluxes, and averaged gap junction ion flux ion fluxes at each holding potential or simulated V_mem_ value. These particular measurements differ from the conventional procedure in its utilization of varying external and internal buffer solution compositions. For example, the role of K^+^ was determined in preliminary experiments by replacing it with N-methyl-D-glucamine in the external buffer solution and comparing control current measurements to measurements under the altered buffer conditions. These current estimates are used downstream in parameterization of $P_{i}$ permeability values for each substituted ion.

### Appendix A.2. Model Core Mathematical Strategy

BETSE utilizes a Voronoi diagram-based cell grid to define cells as irregularly shaped objects, nested within a square environmental grid. This hierarchical arrangement provides flexibility in cell morphology while the grid provides the basis for a Cartesian-like reference coordinate frame. Each cell modeled contains an identifying center point, where scalar parameters (i.e., V_mem,intercellular_ and [ion species]) are defined for the object. The Voronoi diagram-based cell representation allows individual membrane segments to be assigned values for cell perimeter and volume. Furthermore, the membrane segments possess defining tangent and unit vectors at their midpoint, where cell scalar and vector properties are defined. This enables V_mem_ to be defined for each unique membrane segment. The membrane segment midpoints interface with the environmental grid to enable the use of a nearest-neighbor interpolation function. A weighing function counts the number of membrane segments per environmental grid square in a given region and quantitatively assesses a mole fraction for mass transfer between the cell and environment. This is done to prevent the system from violating charge and mass distribution physical laws.

Gradient ($\nabla s_{j}$)—measures amount of change in some property over space at a specific point (j).

Divergence (∇∙${\vec{F}}_{j}$)—measures change in flux (outward or inward) of a vector field from a spatial point (j); essentially assigning each point a designation as a source or sink.

Laplacian ($\nabla^{2}s_{j}$ = ∇∙∇$s_{j}$)—measures divergence of a gradient of some scalar property (relating V_mem_ to ionic charge density via Poisson’s Equation).

Standard finite difference finite volume methods techniques [63] were utilized to solve discrete versions of the divergence, gradient, and Laplacian/inverse Laplacian operators and differential expressions for ion flux on both cell and environmental grids. Then, each core operator was integrated into differential expressions defining bio-electrochemical mass transport. Ion transport was assumed to be influenced by both ion concentration gradients (𝛻c) and local or global voltage potential gradients (𝛻V) in a transport phenomenon known as electrodiffusion. In general, the process can be mathematically characterized via the Nernst-Planck flux expression:

(A1) $$\frac{dc_{i}}{dt}=𝛻\times (D_{i}𝛻c_{i}+\frac{D_{i}z_{i}q}{k_{B}T}𝛻V-\vec{u}c_{i})$$

where $\vec{u}$ represents osmotic fluid flow [64], *q* represents the charge constant for electrons, $k_{B}$ is the Boltzmann constant, and T is the temperature in Kelvins. Alternatively, the GHK flux equation [65] can be utilized to estimate individual ionic transmembrane currents, as shown below:

(A2) $$\Phi_{i}^{mem}=\frac{z_{i}V_{mem}FP_{i,mem}}{RT}\left(\frac{c_{i}^{cell}-c_{i}^{env}exp\;\left(-\frac{z_{i}V_{mem}F}{RT}\right)\;}{1-exp\;\left(-\frac{z_{i}V_{mem}F}{RT}\right)\;}\right)$$

The collective K^+^, Na^+^, and Cl^−^ $\Phi_{i}^{mem}$ voltage-dependent currents, active ion pump flux ($\Phi_{i}^{pump}$), and intercellular gap junction currents ($\Phi_{i}^{gj}$), establish voltage-dependent transmembrane ion flux dynamics, as shown below:

(A3) $$\Phi_{i}^{tot}=\Phi_{i}^{mem}+\Phi_{i}^{gj}+\Phi_{i}^{pump}+\Phi_{i}^{channel}$$

### Appendix A.3. BETSE Parameter and WTC11 Parameter Values and Derivation

#### Appendix A.3.1. GJ Ion Flux parameters

(A4) $$I_{GJ,i}={(P}_{GJ}\times block\%\times \beta_{GJ}^{o})z_{S}^{2}\frac{V_{mem}F^{2}}{RT}\frac{{\left[Ion\right]}_{i}-{\left[Ion\right]}_{i,neighbor}exp(-\frac{z_{S}V_{mem}F}{RT})}{1-exp(-\frac{z_{S}V_{mem}F}{RT})}$$

where block or open % determined by [66] conductance-regulating parameters. Steady-state gap junction conductance is defined below:

(A5) $$g_{SS}=\frac{g_{max}-g_{min}}{1+exp\;[A*\left(V_{j}-V_{0}\right)]\;}+g_{min}$$

where $g_{max/min}$ is the max/min value that can be reached given high $V_{intercell}$, $g_{j}$ is gap junction instantaneous series conductance, $V_{0}$ is the voltage at which conductance is halfway between its’ minimum and maximum values, $g_{SS}$ represents steady-state conductance value, and A is the constant for voltage sensitivity.

(A6) $$g_{j}=g_{SS}+{(g}_{0}-g_{SS})exp(-t/\tau )$$

where τ is a time constant and $g_{0}$ is the initial conductance value of the gap junctions. Normalized steady-state conductances were calculated by manipulating open and close rates α and β.

(A7) $$\alpha =\lambda *exp\;\left[-A_{\alpha }\times \left(V_{j}-V_{0}\right)\right]$$

(A8) $$\beta =\lambda *exp\left[A_{\beta }\times \left(V_{j}-V_{0}\right)\right]$$

$A_{\alpha /\beta }$ represent open and close voltage sensitivities, λ represents a magnitude scaling parameter, α/β represent the channel open/close rates, and β is set to asymptotically approach a maximum value of 20/sec, which prevents a situation in which calculated current pulse responses exponentially increase during terminal phases from occurring.

(A9) $$\beta^{\prime }=\frac{\beta }{1+50\beta };$$

In accordance with experimental data provided from [41], we iteratively simulated 25 and 75% cell-cell ion flux inhibition (or more simply put relative channel block strength) conditions. Furthermore, we set voltage sensitivity parameters in accordance with Cx43 (GJA1) levels from [28]. The Boltzmann sigmoid used to represent voltage-dependent steady-state gating is fit to the [66] representation (Equations (A4)–(A13)).

#### Appendix A.3.2. β-GA Effect Estimation

The addition of β-GA was approximated by a 25 or 75% magnitude reduction in cell-cell ionic conductance; there is no observed change to the shape of the bell curve defining transjunctional voltage-dependent current transfer, so the only parameter changed from the initial settings is the surface area as a fraction of cell membrane surface area ($\beta_{GJ}^{0}$). The original value (1 × 10^−7^) was adjusted to 25 or 75% of the difference between the original value and the completely closed value (1 × 10^−9^).

25% magnitude reduction:

(A10) $$\left[\left(1\times 10^{-7}\right)-\left(1\times 10^{-9}\right)\right]*\left(0.75\right)=7.425\times 10^{-8}$$

(A11) $$\left(1\times 10^{-9}\right)+\left(7.425\times 10^{-8}\right)=\beta_{GJ}^{o}=7.525\times 10^{-8}$$

75% magnitude reduction:

(A12) $$g_{j}=g_{SS}+{(g}_{0}-g_{SS})exp(-t/\tau )$$

(A13) $$\left(1\times 10^{-9}\right)+\left(2.475\times 10^{-8}\right)=\beta_{GJ}^{o}=2.575\times 10^{-8}$$

#### Appendix A.3.3. Single-Ion Flux Parameters

(A14)
$$I_{i}=P_{S}z_{S}^{2}\frac{V_{mem}F^{2}}{RT}\frac{{\left[S\right]}_{i}-{\left[S\right]}_{o}exp(-\frac{z_{S}V_{mem}F}{RT})}{1-exp(-\frac{z_{S}V_{mem}F}{RT})}$$

The permeability is the adjustable parameter used to control voltage-dependent ionic flux; these values have been matched to our in-house whole-cell patch clamp data.

#### Appendix A.3.4. Na^+^/K^+^-ATPase Parameters

The V_mem_-dependent Na^+^/K^+^-ATPase pump rate is estimated, based on the previous characterization by [30].

The Na^+^/K^+^-ATPase pump Gibbs free energy ($∆G_{pump}$) formula is shown below:

(A15) $$∆G_{pump}=∆G_{ATP}^{0}+RTlnln\;\left(Q\right)-FV_{mem}$$

where $∆G_{ATP}^{0}=-36e^{3}\frac{J}{mol}$ represents standard free energy for the ATP hydrolysis reaction, Q represents the reaction quotient, and F is Faraday’s constant.

(A16) $$Q=\frac{\left[{ADP}_{cell}\right]\left[{Pi}_{cell}\right]{\left[Na_{env}\right]}^{3}{\left[K_{cell}\right]}^{2}}{\left[ATP_{cell}\right]{\left[Na_{cell}\right]}^{3}{\left[K_{env}\right]}^{2}}$$

where $Na_{env}$ and $Na_{cell}$ represent cell and environmental sodium concentrations, $K_{env}$ and $K_{cell}$ represent cell and environmental sodium concentrations, and ${ADP}_{cell}$ and ${ATP}_{cell}$ as cell ADP and ATP concentrations;

(A17) $$K_{NaKATP}^{eqm}=exp\;\left(\frac{-∆G_{ATP}^{0}+FV_{mem}}{RT}\right)$$

where $K_{NaKATP}^{eqm}$ represents the Na^+^/K^+^-ATPase reaction equilibrium constant;

(A18) $$\Phi_{NaKATP}=\Phi_{NaKATP}^{max}\left(r_{f}-\frac{Q}{K_{eqm}}r_{r}\right)$$

where $\Phi_{NaKATP}^{max}$ represents the max pump rate, which we estimated from the values provided in [30], $r_{f}$ represents forward reaction pump rate constant, and $r_{r}$ represents reverse reaction pump rate constant.

(A19) $$r_{f}=\frac{\frac{cATP}{K_{ATP}}{\left(\frac{cNa_{in}}{K_{Na}}\right)}^{3}{\left(\frac{cK_{out}}{K_{K}}\right)}^{2}}{\left(1+\frac{cATP}{K_{ADP}}\right)\left(1+{\left(\frac{cNa_{in}}{K_{Na}}\right)}^{3}\right)\left(1+{\left(\frac{cK_{out}}{K_{K}}\right)}^{2}\right)}$$

where $K_{K}=0.2\;mol/m^{3}$, $K_{Na}=5\;mol/m^{3}$, $K_{Cl}=1.0\;mol/m^{3}$ represent chloride, potassium, and sodium rate constants.

(A20) $$r_{r}=\frac{\frac{\left[ADP\right]}{K_{ADP}}\frac{\left[Pi\right]}{K_{Pi}}{\left(\frac{\left[Na_{out}\right]}{K_{Nao}}\right)}^{3}{\left(\frac{\left[K_{in}\right]}{K_{Ki}}\right)}^{2}}{\left(1+\frac{\left[ADP\right]}{K_{ADP}}\right)\left(1+\frac{\left[Pi\right]}{K_{Pi}}\right)\left(1+{\left(\frac{\left[Na_{out}\right]}{K_{Nao}}\right)}^{3}\right)\left(1+{\left(\frac{\left[K_{in}\right]}{K_{Ki}}\right)}^{2}\right)}$$

where $K_{K}=0.2\;mol/m^{3}$, $K_{Na}=5\;mol/m^{3}$, $K_{ATP}=0.15\;mol/m^{3}$, $K_{ADP}=1.0\;mol/m^{3}$, $K_{Pi}=1.0\;mol/m^{3}$, $K_{Nao}=140.0\;mol/m^{3}$, $K_{Ki}=120.0\;mol/m^{3}$ represent reaction rate constants [29].

WTC11 hiPSC Parameters

hiPSC Ionic Profile 1:

$$D_{mem_{K^{+}}}=1.274\times 10^{-17}\;m^{2}/s\;$$

$$D_{mem_{{Na}^{+}}}=4.478\times 10^{-18}\;m^{2}/s\;$$

$$D_{mem_{{Cl}^{-}}}=1.973\times 10^{-19}\;m^{2}/s$$

hiPSC Ionic Profile 2:

$$D_{mem_{K^{+}}}=1.274\times 10^{-17}\;m^{2}/s$$

$$D_{mem_{{Na}^{+}}}=4.478\times 10^{-18}\;m^{2}/s\;$$

$$D_{mem_{{Cl}^{-}}}=1.973\times 10^{-19}\;m^{2}/s$$

#### Appendix A.3.5. WTC11-Specific Voltage Gated K^+^ Ion Channel Parameters

The ionic permeability was directly regulated by V_mem_; therefore, we fit $P_{i}^{channel}$ (or equivalent K^+^ ion channel gating parameter, $g_{k}$) to the difference between measure patch-clamp-derived transmembrane ion current density and model-estimated transmembrane current density without K^+^ ion flux.

(A21) $$P_{i}^{channel}\sim g_{k}=(m_{\infty }-m)/(m_{\tau })$$

where

(A22) $$m_{\infty }=\frac{1}{1+\exp\left(\frac{V_{mem}-\left(-30\right)}{-14.7}\right)}$$

(A23) $$m_{\tau }=\frac{1}{1+\exp\left(\frac{V_{mem}-\left(-46.56\right)}{-44.14}\right)}$$

$$g_{K^{+},max}=6.71\times 10^{-16}\;\frac{m^{2}}{s}$$

### Appendix A.4. Main Model Parameters

**Table A1:** Parameter values used in computational model.

Parameter	Description	Typical Value	Units
i	Ion index (i = Na, K, Cl, Ca, H, M)		
$D_{o_{i}}$	Free diffusion coefficient for ion i	$1.0*10^{-9}$	$\frac{m^{2}}{s}$
t	Time	1–10	s
x, y	Spatial coordinates	500	μm
h	System height	10	μm
$\Delta G_{ATP}^{o}$	Standard free energy of ATP hydrolysis	37	kJ/mol
T	Temperature	310	K
F	Faraday’s constant	96,485	C/mol
R	Ideal gas constant	8.3145	J/K mol
q	Electron charge constant	$1.6*10^{-19}$	C/ion
$k_{b}$	Boltzmann constant	$1.38*10^{-23}$	J/K
$v_{cell},\;v_{ecm}$	Cell and extracellular	$7.85*10^{-16}$	$m^{3}$
$\sigma_{cell},\;\sigma_{mem},\;\sigma_{ecm}$	Cell membrane and extracellular surface area	$3.14*10^{-10}$	$m^{2}$
$c_{mem}$	Membrane capacitance	0.022	F/$m^{2}$
$c_{self}$	Electrolyte-induced self-capacitance	0.86	F/$m^{2}$
$\alpha_{pump}$	Max rate constant for pump	$2.0*10^{-7}$	1/s $m^{2}$
$v_{\frac{1}{2}GJ}\;$	GJ voltage-gating half-closed parameter	50	mV
$d_{mem}$	Cell membrane thickness	$7.5*10^{-9}$	m
$d_{gj}$	Intercellular spacing	$26.0*10^{-9}$	m
μ	Water viscosity	$5.0*10^{-3}$	Pas
**Variable**	**Description**	**Typical value**	**Units**
$c_{i_{ext}}$	Extracellular concentration	1–150	$\frac{mol}{m^{3}}$
$c_{i_{int}}$	Intracellular concentration	1–150	$\frac{mol}{m^{3}}$
$D_{mem_{i}}$	Membrane diffusion coefficient for ion i	$1.0*10^{-18}$	
$P_{mem_{i}}$	Membrane permeability for ion i	0.13	nm/s
$V_{cell},\;V_{env}$	Voltage in cell and environment	−10 to −80	mV
$V_{mem}$	Cell transmembrane voltage potential	−10 to −80	mV
$\vec{\Phi_{i}}$	Mass flux of ion i	1.0	$\frac{\mu mol}{s-m^{2}}$
$\rho_{e}$	Ionic charge density	600	$\frac{\mu mol}{s-m^{2}}$
$\vec{J}$	Ionic current density	10–500	$\frac{C}{m^{3}}$
$\beta_{GJ}^{o}$	GJ diffusion scaling-coefficient	$5.0*10^{-7}$	
$\beta_{TJ}$	TJ diffusion scaling-coefficient	$1.0*10^{-7}$	
$D_{NaV},\;D_{KV}$	Max membrane diffusion for voltage-gated channel	$1.0*10^{-14}$	$\frac{m^{2}}{s}$
$\vec{E}$	Electric field	$1.0*10^{5}$	V/m

## Appendix B

### Appendix B.1. Quad-Tree Analysis

We follow the analysis of an image into Quad-Trees according to Libby, Briers et al. (2018) [6] with changes from a colored image into greyscale. An input n x n greyscale input photo is read into Matlab as a matrix $A_{kxk}$ where each element $a_{ij}=<a_{ij}^{\left(Gr\right)}>$ is the normalized RGB pixel value for a specific <i,j> coordinate.

$$0\leq a_{ij}^{\left(Gr\right)}\leq 1$$

As described in [6], given an input image’s network A, the submatrix A[i_s_, i_e_, j_s_, j_e_] corresponds to the rows and columns that are ½ the original length to create four quadrants from the original network. Following this syntax, we represented matrix A as a quad-tree hierarchical data structure Q=(V,R) where each vertex v∈V represents a quad-tree submatrix and the relation R⊂VxV defines four children for each specified vertex. Additionally, each edge of the tree is labeled with the direction of the submatrix created represented as the child, using directions north west (NW), north east (NE), south east (SE), and south west (SW).

A representation function $\mu^{\left(c\right)}\left(v\right):V\to \left[0,b\right]\;x\;[0,b]$ is defined for each matrix and submatrix, where *b* defines the image coordinate boundaries used in image/matrix quad-tree partitions. The function $\mu^{\left(c\right)}\left(v\right)$ provides the mean value and variance for the concentration of pixel greyscale intensity values in a particular region of the space represented by the quad-tree vertex, v.

(A24) $$\mu^{\left(c\right)}\left(v\right)=\left(\mu_{1}^{\left(c\right)},\mu_{2}^{\left(c\right)}\right)$$

(A25) $$\mu_{1}^{\left(c\right)}=\frac{1}{\left(i_{e}-i_{s}+1)(j_{e}-j_{s}+1\right)}\sum_{i,j\in \left\{i_{s},\ldots ,i_{e}\right\}\;x\;\{j_{s},\;\ldots ,\;j_{e}\}}a_{ij}^{\left(c\right)}$$

(A26) $$\mu_{2}^{\left(c\right)}=\frac{1}{\left(i_{e}-i_{s}+1)(j_{e}-j_{s}+1\right)}\sum_{i,j\in \left\{i_{s},\ldots ,i_{e}\right\}\;x\;\{j_{s},\;\ldots ,\;j_{e}\}}{\left(a_{ij}^{\left(c\right)}-\mu_{1}^{\left(c\right)}\right)}^{2}$$

The mean and variance metrics $\mu_{1}^{\left(c\right)}$ and $\mu_{2}^{\left(c\right)}$ provide information on greyscale intensity within a particular image frame and variance across the current level of resolution.

We assumed a relaxed partitioning requirement that allows each quad-tree submatrix to possess non-equal values while maintaining the ability to develop into children of a node. Also, we limited each quad-tree to a depth of 5 to prevent exponential growth of the quad-tree vertices.

The quad-tree variance function computes the change in quantitative valuation between parent and child nodes in each network. However, the ‘change in distance’ here refers to spatial distance changes within a level and not between levels of resolution depth.

### Appendix B.2. QTS/TSSL Formula and Pattern Classification

Quadtrees recursively partition two-dimensional spatial regions of an image into quadrants of increasing levels of spatial resolution [47]. These partitions specifically contain pixel region-specific intensity information, which would biophysically correspond to a spatial concentration and relative variance of DiBAC_4_(3) fluorescence (experimental) or relatively depolarized BETSE-estimated V_mem_ (computational). Tree Superposition Spatial Logic (TSSL) utilizes formal language pattern descriptors, or pattern classifiers, to define local and global pattern features of an image or image set. TSSL is powerful for quantitatively capturing complexity in images at different scales and therefore useful here in identifying pockets of ion flow and regimes of isolated cells within the iPSC colony. Generated quad transition systems were translated into condition-specific classifier rules to train a supervised machine learning algorithm, RIPPER [67], and these condition-specific rules served as the means for quantitative pattern similarity comparison across simulated culture conditions, as well as comparison to their experimental condition analogs. RIPPER compares images comprising the desired target pattern type (+) against the remaining simulated image sets (−) to determine descriptive ‘rule sets’ for each culture condition-specific output image set. Each rule set is a tuple of translated TSSL formulae, comprised of formal language descriptors of multiresolution patterns features present in (+) images; interpreted as nested if-else statements, they allow spatial features on bioelectric patterning (i.e., iso-potential cell clusters or highly heterogeneous V_mem_ patterns) to be identified and stored for downstream image pattern comparison. Once a desired pattern type is identified, RIPPER enables quantitative similarity assessment for input images representing differing culture condition patterns outcomes Input experiment or simulated images were assessed a similarity score ranging from −1 to +1 based on how similar or dissimilar they are to the target pattern, with +1 indicating perfect match between target and image.

The defining algorithm qualitative and quantitative semantics is briefly described below and can be reviewed in further detail in [47,68]. Quadtrees *Q_TS_* are defined as:

Syntax: $Q_{TS}=(S,s_{l},\tau ,\Sigma ,\left[.\right],L)$

(1) S is a set of states, $s_{l}$ being the initial state;

(2) τ is the transition relation;

(3) Σ is the finite set of variables;

(4) [.] is the labeling function;

(5) L is a labeling function defining the set of directions associated with each state transition $L:\;\tau \to 2^{D}$. It possesses the properties $D=\left\{NW,NE,SE,SW\right\}$ and $\forall \left(s,t\right),\left(s,t^{\prime }\right)\in \tau ,$ with $t\neq t^{\prime }$, and $L\left(s,t\right)\cap L\left(s,t^{\prime }\right)=\emptyset ,\;\forall i\;\in \;N$.

This syntax provides a more compact representation of the original quad-tree for image comparison quantification and TSSL formula extraction. The QTS building algorithm starts from an input quad-tree Q=(V,R) of a matrix $A_{kxk}$ and a unique labeling function LQ:R→D. The algorithm is constructed such that it provides a compact labeling function tuple that characterizes the hierarchical connectivity amongst quad-tree nodes. The initialization phase defines structures for quad-tree states, directions, variables, and state transitions with subsequent equivalent leaf set partition. A self-transition is defined for each state in the partitioned set and non-leaf child nodes were assigned a node transition. The parent to child transition is denoted a_1_(t), etc. to denote the directional transitions. Then, all non-leaf nodes are explored in a breadth-first search and new state tuples are added to the QTS structure. The same equivalent quad-tree node aggregation procedure defined earlier is repeated for the non-leaf nodes.

The TSSL syntax is as follows:

$$\varphi \;∷=⏉\;\left|\;⊥\right|\;m∼d\;\left|\;\neg \varphi \;\right|\;\varphi_{1}\;\wedge \;\varphi_{2}\;\left|\;\exists_{B}\circ \;\varphi \;\right|\;\forall_{B}\;\circ \;\varphi \;\left|\;\exists_{B}\varphi_{1}U_{k}\varphi_{2}\;\right|\;\exists_{B}\varphi_{1}U_{k}\varphi_{2}$$

with $\sim \in \left\{\leq ,\geq \right\},d\in \left[0,b\right],b\in R_{+},k\epsilon N_{>0},B\subseteq D:B\neq \emptyset ,$ and m∈Σ, with being the set of variables. From this specified syntax, two additional spatial mathematical operators can be defined: the exist eventually operator $\exists_{B}F_{k}$, the for all eventually operator $\forall_{B}F_{k}$, the exists globally operator $\exists_{B}G_{k}$, and the for all globally operator $\forall_{B}G_{k}$. This defined syntax can also be represented as shown below:

$$\exists_{B}F_{k}\varphi \;:=\exists_{B}⏉U_{k}\varphi \;\exists_{B}G_{k}\varphi \;:=\neg \forall_{B}F_{k}\neg \varphi .$$

$$\forall_{B}F_{k}\varphi \;:=\forall_{B}⏉U_{k}\varphi \;\forall_{B}G_{k}\varphi \;:=\neg \exists_{B}F_{k}\neg \varphi .$$

WEKA JRipper and Naïve Bayes classification algorithms were utilized to calculate correct pattern identification rate via 10-fold stratified cross validation; specifically, 10-fold stratified cross validation was performed using the ‘NaïveBayes’ classification module and batch size = 100; the pattern F-measure, precision, recall, and precision-recall curve statistics were also evaluated with the ‘NaiveBayes’ module. JRipper, a propositional rule learner-based algorithm, was used to generate BETSE pattern classifiers and estimate correct pattern classification rate from provided training data. In this study, each target BETSE culture condition-specific pattern image set was identified as the (+) images and the image sets pertaining to the other remaining four conditions were denoted as (−). This process was repeated for each of the simulated condition image sets, including the ‘GJ 50/50 Mosaic’ and ‘GJ 100% decrease’ pattern classes.

### Appendix B.3. Similarity Score Calculations

Normalization of similarity score data was performed in the scikit-learn Python package ‘minmax_scale’, where numerical features (similarity scores) were scaled such that each feature fell between a given range, defined by the minimum and maximum score values, were transformed to values between 0 and 1. This process was performed for each in vitro condition image set comparison condition similarity comparison score set with respect to BETSE pattern classifiers. This transformation was done to ease visualization for the reader and to enable quick assessment of meaningful pattern similarity score differences between classes. Outliers to be excluded from violin plots were chosen via manual set z-score distribution inspection; these scores were calculated with the Matlab ‘zscore’ function; Welch’s *t*-test was utilized to assess statistical significance between sample similarity score distributions with unequal sizes and variances with the Matlab ‘ttest2’ function.
