# Supplementary material for: Identification of Distinct, Quantitative Pattern Classes from Emergent Tissue-Scale hiPSC Bioelectric Properties
Source: Cells. 2024 Jul 2;13(13):1136. doi: 10.3390/cells13131136 (PMC11240333; doi:10.3390/cells13131136)
Supplement: Supplementary file 1 [file cells-13-01136-s001.zip › cells-3029497-supplementary.pdf]

Supplementary text: **TSSL syntax describing each pattern classifier:**

[1] 'Ctrlcomp' pattern classifier (n = 200).

$$\phi_{Control} = \forall_{\{Root\}} \circ [0.7726 \leq m \leq 0.7196] \wedge \forall_{\{NW,NE,SE,SE,SE\}} \circ [m \geq .705882] \wedge \forall_{\{NW,NE,NE\}} \circ [m \geq .7033] \wedge \forall_{\{NW,SW,NW,SW,SW\}} \circ [m \leq .8237] \wedge \forall_{\{NW,SW,NW,SW,SE\}} \circ [m \geq .7569] \wedge \forall_{\{NW,SE,NW\}} \circ [m \leq .9115] \wedge \forall_{\{NW,SE,NE,SE\}} \circ [m \geq .6542] \wedge \forall_{\{NW,SE,NE,SE,SE\}} \circ [m \leq .9176] \wedge \forall_{\{NW,NW,SW,NW,SW\}} \circ [m \leq .7794] \wedge \forall_{\{NW,SE,SW,NE,SW\}} \circ [m \leq .9118] \wedge \forall_{\{NW,SW,SW,NW,SW\}} \circ [m \leq .9111] \wedge \forall_{\{NW,SE,NE,NW\}} \circ [m \leq .9297] \wedge \forall_{\{NW,SE,SE\}} \circ [m \geq .6955] \wedge \forall_{\{NW,SE,SE,NE,SW\}} \circ [m \leq .9229] \wedge \forall_{\{NW,NE,NE,NE,NW\}} \circ [m \geq .5071] \wedge \forall_{\{NW,NE,NW,NE,SE\}} \circ [m \leq .9292]$$

[2] 'GJ75comp' pattern classifier (n = 200).

$$\phi_{GJ,75\%\ Decrease} = \forall_{\{Root\}} \circ [0.7238 \geq m] \wedge \forall_{\{NW,NW,NW,SE,SE\}} \circ [m \geq .9020] \wedge \forall_{\{NW,NW,NW,NE,NE\}} \circ [m \leq .7559] \wedge \forall_{\{NW,NE,SW,SW,SW\}} \circ [m \leq .6275] \wedge \forall_{\{NW,NE,NW,SW,SW\}} \circ [m \geq .9059] \wedge \forall_{\{NW,NW,NE,SE,SE\}} \circ [m \leq .6275] \wedge \forall_{\{NW,NE,SW,SW,NE\}} \circ [m \geq .8825] \wedge \forall_{\{NW,SE,NE\}} \circ [m \leq .8671] \wedge \forall_{\{NW,SE,NW,SW,NE\}} \circ [m \leq .6256] \wedge \forall_{\{NW,SE,NW,NW,NE\}} \circ [m \geq .8814] \wedge \forall_{\{NW,NE,SW,SW,NE\}} \circ [m \geq .6431] \wedge \forall_{\{NW,SE,NW,SE,SE\}} \circ [m \geq .8902] \wedge \forall_{\{NW,NE,SE,NW\}} \circ [m \geq .7015] \wedge \forall_{\{NW,SE,NE,NE,NE\}} \circ [m \leq .5498] \wedge \forall_{\{NW,NE,SE,NE,SE\}} \circ [m \leq .4814] \wedge \forall_{\{NW,NW,SW,NE,NE\}} \circ [m \leq .4588] \wedge \forall_{\{NW,NE,SE,SE,SE\}} \circ [m \geq .9451] \wedge \forall_{\{NW,SE,NW,NW,NE\}} \circ [m \leq .5765] \wedge \forall_{\{NW,SE,NW,SW,SW\}} \circ [m \geq .8912] \wedge \forall_{\{NW,SE,NE,NE,NW\}} \circ [m \leq .5609] \wedge \forall_{\{NW,NE,SE,NE,NE\}} \circ [m \geq .8720] \wedge \forall_{\{NW,NE,NE,NE,SW\}} \circ [m \leq .5706] \wedge \forall_{\{NW,NE,SW,NW,NE\}} \circ [m \geq .9490]$$

[3] 'GJ25comp' pattern classifier (n = 200).

$$\phi_{GJ,25\%\ Decrease} = \forall_{\{NW,SE,NE,SE\}} \circ [m \leq 0.1255] \wedge \forall_{\{NW,NW,SW,SW,SE\}} \circ [m \geq .3304] \wedge \forall_{\{NW,NE,NE,SE,NW\}} \circ [m \leq .2510] \wedge \forall_{\{NW,SE,NE,SE\}} \circ [m \leq .1240] \wedge \forall_{\{NW,NW,NE,NE\}} \circ [m \geq .1794] \wedge \forall_{\{NW,SW,NW,SW,NE\}} \circ [m \geq .3402] \wedge \forall_{\{NW,SW,SW,SW\}} \circ [m \leq .1387] \wedge \forall_{\{NW,NE,SE\}} \circ [m \leq .6460]$$

[4] 'K10mMcomp' pattern classifier (n = 200).

$$\phi_{K^+\ 10\ mM} = \forall_{\{Root\}} \circ [m \geq 0.4552] \wedge \forall_{\{NW,NW,NW,NE\}} \circ [m \leq 0.1637] \wedge \forall_{\{NW,SW,NW,NW,SE\}} \circ [m \leq 0.2510] \wedge \forall_{\{Root\}} \circ [m \geq 0.6986] \wedge \forall_{\{NW,SE,SE,NE\}} \circ [m \leq 0.7272] \wedge \forall_{\{NW,SE,SE,SW,NE\}} \circ [m \geq 0.8882] \wedge \forall_{\{NW,NW,SW,SE,NW\}} \circ [m \geq 0.7765] \wedge \forall_{\{Root\}} \circ [m \geq 0.7152] \wedge \forall_{\{NW,NW,NW,NW,NE\}} \circ [m \leq 0.4755] \wedge \forall_{\{NW,NE,SE,NE,NE\}} \circ [m \leq 0.6961] \wedge \forall_{\{NW,NE,NW,SW,SW\}} \circ [m \geq 0.8108]$$

[5] 'K10mMcomp' pattern classifier (n = 200).

$$\phi_{K^+\ 20\ mM} = \forall_{\{NW,NW,SE,SE,SW\}} \circ [m \geq 0.9235] \wedge \forall_{\{NW,NW,SE,SE,NW\}} \circ [m \leq 0.8971] \wedge \forall_{\{NW,NW,SE,NE,SW\}} \circ [m \geq 0.6255] \wedge \forall_{\{NW,SE,NW,NW,SE\}} \circ [m \geq 0.9225] \wedge \forall_{\{NW,SE,NW,NW,NE\}} \circ [m \leq 0.9049] \wedge \forall_{\{NW,NW,NW,NE\}} \circ [m \geq 0.1728] \wedge \forall_{\{NW,SE,NE,NW,SW\}} \circ [m \geq 0.9569] \wedge \forall_{\{NW,NW,NE,NE,SE\}} \circ [m \geq 0.5863] \wedge \forall_{\{NW,NE,SW,SE\}} \circ [m \leq 0.9206] \wedge \forall_{\{NW,SW,SW,SW,NE\}} \circ [.0745 \leq m \leq 0.0824] \wedge \forall_{\{NW,SW,SE,SW\}} \circ [m \leq 0.1252] \wedge \forall_{\{NW,NW,SE,SW,NE\}} \circ [m \geq 0.9431] \wedge \forall_{\{NW,SE,NW,SE,NE\}} \circ [m \geq 0.9029] \wedge \forall_{\{NW,SE,NW,NE,SE\}} \circ [m \leq 0.9225] \wedge \forall_{\{NW,SW,SW,NW,NE\}} \circ [m \geq 0.9912]$$

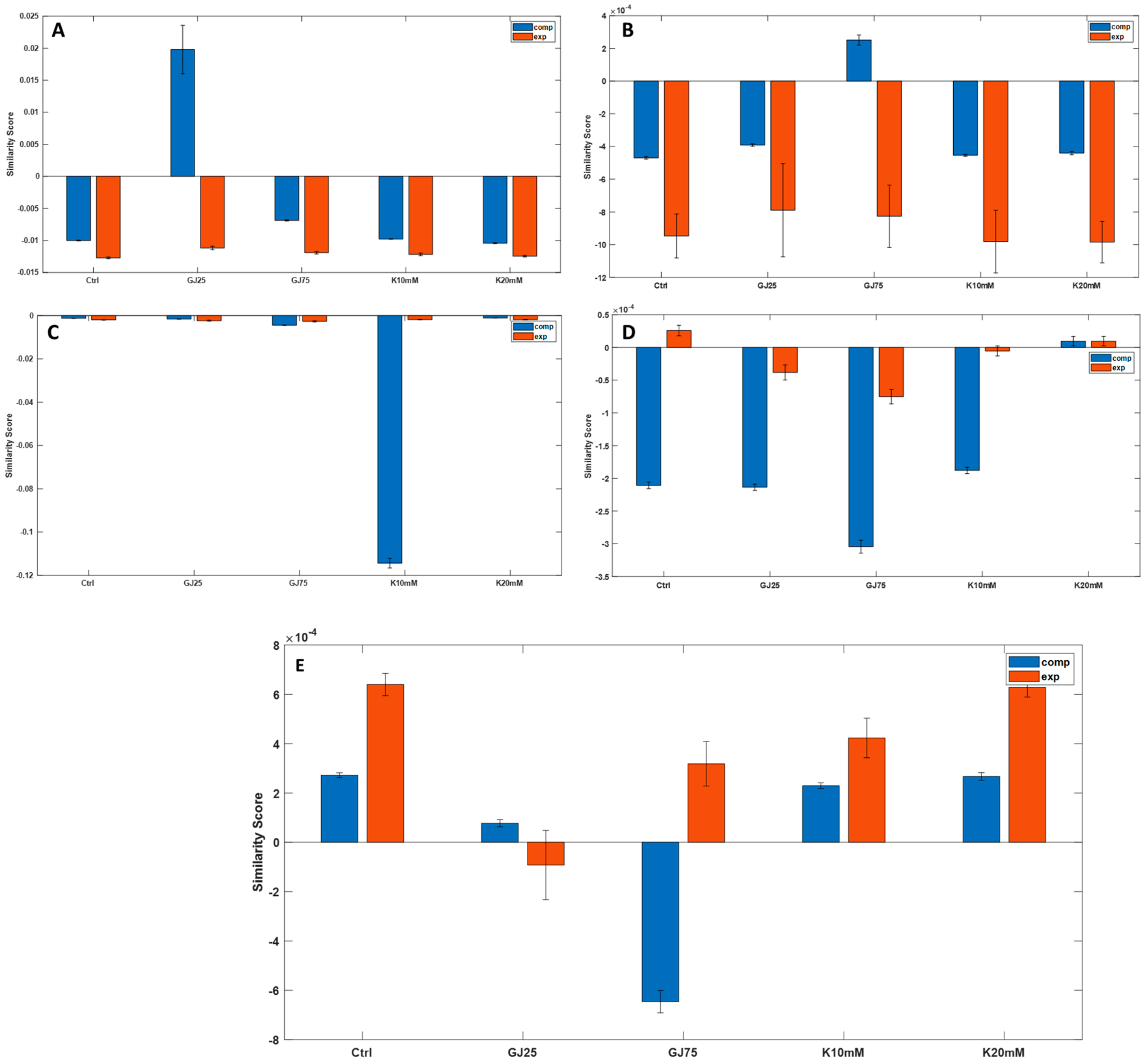

Figure S1. Similarity scores are shown for comparisons between BETSE culture condition target pattern classifiers. (**A**) Simulated ‘GJ25comp’ classifier quantitative comparison to other BETSE and in vitro condition-specific image patterns. (**B**) Simulated ‘GJ25comp’ classifier quantitative comparison to other BETSE and in vitro condition-specific image patterns. (**C**) Simulated ‘K10mMcomp’ classifier quantitative comparison to other BETSE and in vitro condition-specific image patterns. (**D**) Simulated ‘K20mMcomp’ classifier quantitative comparison to other BETSE and in vitro condition-specific image patterns. (**E**) Simulated 'Ctrlcomp‘ classifier quantitative comparison to other BETSE and in vitro condition-specific image patterns. Condition nomenclature: ‘Ctrlcomp’ = Un-modified culture media, simulated patterns; ‘GJ25comp’ = Weak inhibition of gap junction cell-cell communication, simulated patterns; ‘GJ75comp’ = Strong inhibition of gap junction cell-cell communication, simulated patterns; ‘K10mMcomp’ = 10 mM KCl culture media supplement, simulated patterns; ‘K20mMcomp’ = 20 mM KCl culture media supplement, simulated patterns.

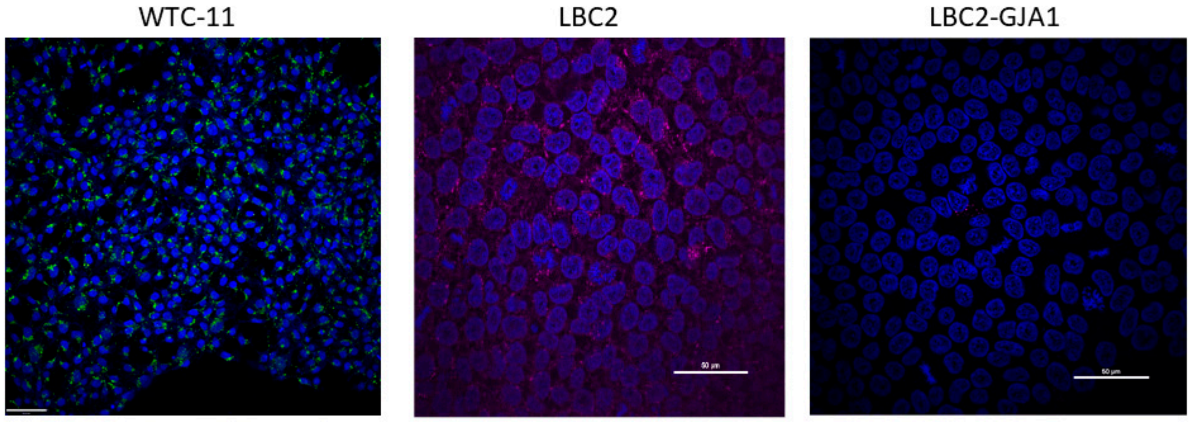

Figure S2. Figure S2. Expression of Connexin 43 in hiPSC clusters, as indicated by Cx43 (GJA1) immunocytochemical staining. Left: WTC11 clusters, Cx43 (GJA1) is shown in green and the nuclear Hoechst33342 stain is shown in blue. Middle: LBC2 hiPSC clusters (non-silenced), Cx43 (GJA1) is shown in pink and the nuclear Hoechst33342 stain is shown in blue. Right: LBC2-GJA1 hiPSC clusters upon stable doxycycline-induced gene knockout, Cx43 (GJA1) is shown in pink and the nuclear Hoechst33342 stain is shown in blue.

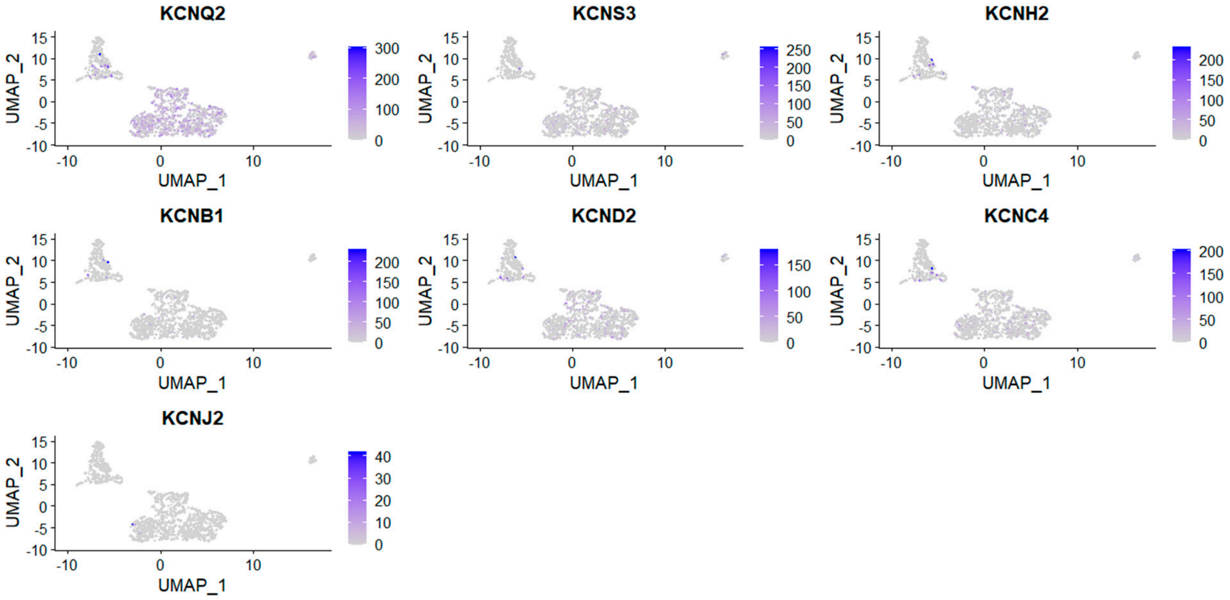

Figure S3. Single-cell RNA sequencing expression profiles are shown for: voltage-gated K+ ion channel isoforms Kv7.2 (KCNQ2), Kv9.3 (KCNS3), Kv11.1 (KCNH2), Kv2.1 (KCNB1), Kv4.2 (KCND2), Kv3.4 (KCNC4), and Kir2,1 (KCNJ2); voltage-gated Na+ ion channel isoforms Nav1.6 (SCN8A), and Nav1.9 (SCN11A); Na+/K+-ATPase α (ATP1A1) and β (ATP1B1) subunit isoforms, and GAPDH in WTC11 hiPSCs.

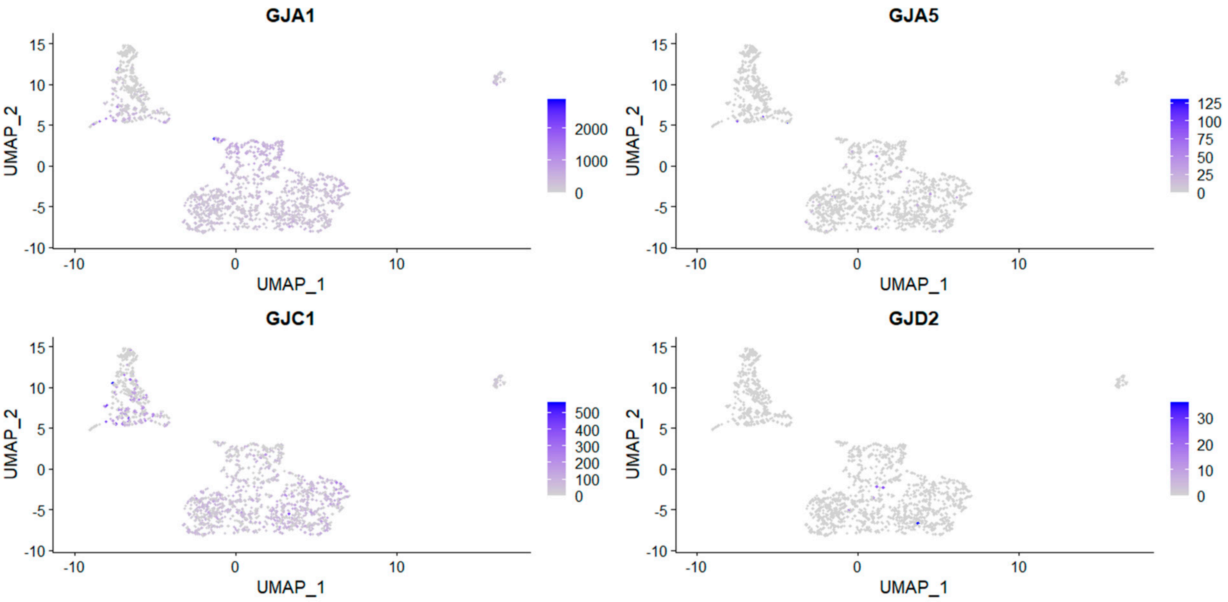

Figure S4. Single-cell RNA sequencing expression profiles are shown for: Cx40 (GJA5), Cx43 (GJA1), Cx36 (GJD2), and Cx45 (GJC1) connexin isoforms in WTC11 hiPSCs.

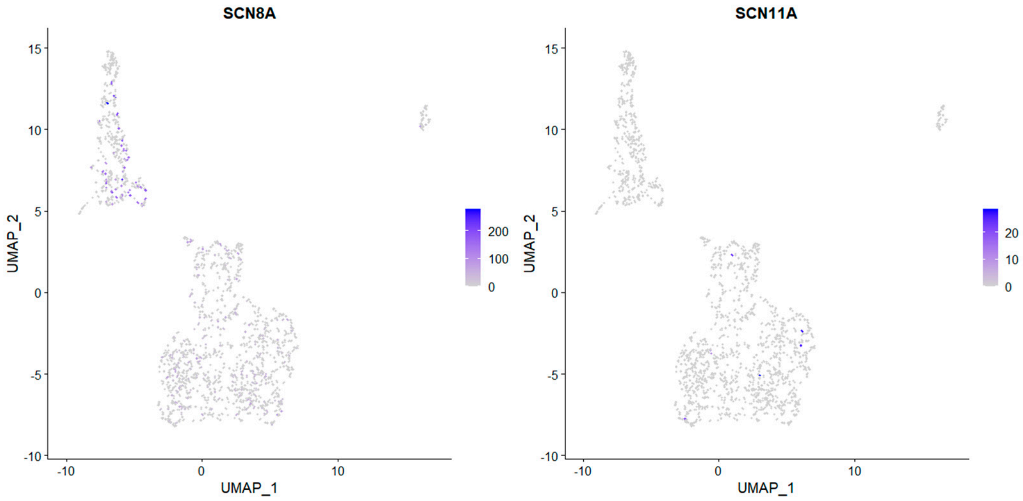

Figure S5. Single-cell RNA sequencing expression profiles are shown for: voltage-gated Na^+ ion channel isoforms Nav1.6 (SCN8A), and Nav1.9 (SCN11A) in WTC11 hiPSCs.

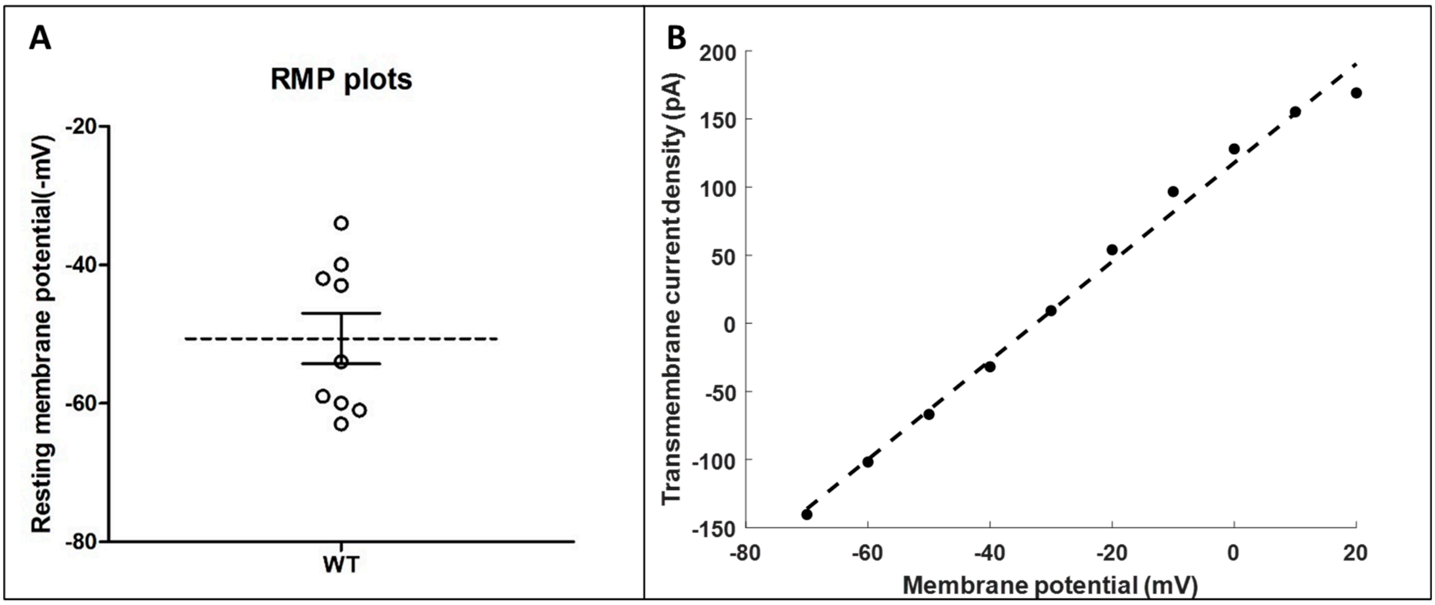

Figure S6. Whole-cell patch clamp data is shown for (**A**) individual samples (n=9) are patched at their respective resting membrane voltage potentials (V_{mem}); (**B**) transmembrane current density in response to increasing steady-state electrode holding potentials (mV); the dark line represents linear fit (R^2 = 0.9729) between the holding voltages and whole-cell transmembrane current density; the derived curve was used for downstream ionic flux parameterization.

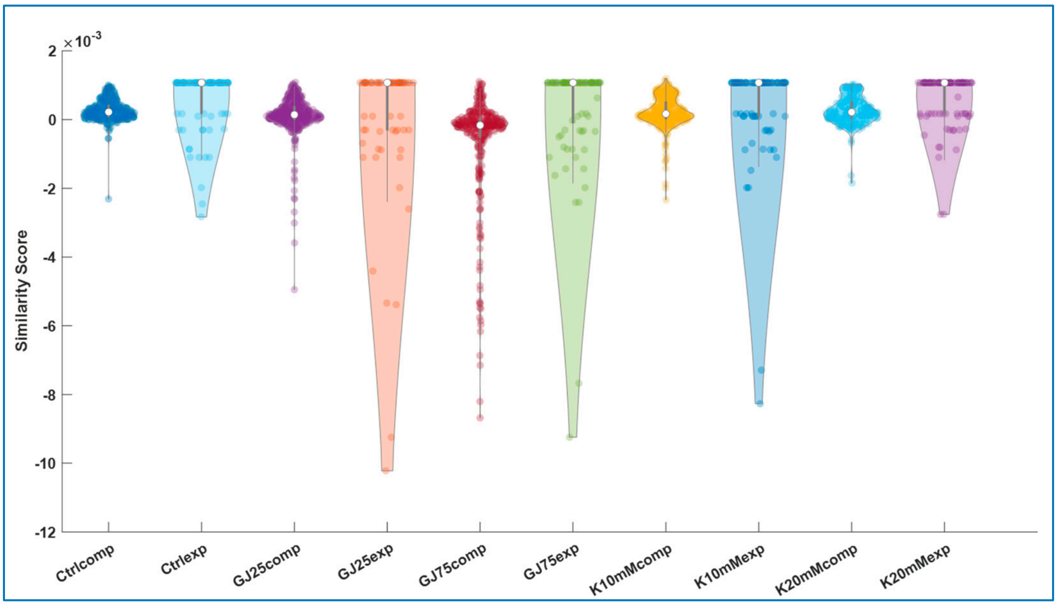

Figure S7. Violin plot of the similarity score distribution for each image set in comparison to the simulated 'Ctrlcomp' target pattern profile. Condition nomenclature: 'Ctrlcomp' = Un-modified culture media, simulated patterns; 'GJ25comp' = Weak inhibition of gap junction cell-cell communication, simulated patterns; 'GJ75comp' = Strong inhibition of gap junction cell-cell communication, simulated patterns; 'K10mMcomp' = 10 mM KCl culture media supplement, simulated patterns; 'K20mMcomp' = 20 mM KCl culture media supplement, simulated patterns; 'Ctrlexp' = Un-modified mTeSR culture media, experimentally-observed patterns; 'GJ25exp' = mTeSR culture media supplemented with 10 uM 18β-GA (gap junction inhibitor), experimentally-observed patterns; 'GJ75exp' = mTeSR culture media supplemented with 60 uM 18β-GA (gap junction inhibitor), experimentally-observed patterns; 'K10mMexp' = 10 mM KCl mTeSR culture media supplement, experimentally-observed patterns; 'K20mMexp' = 20 mM KCl mTeSR culture media supplement, experimentally-observed patterns.

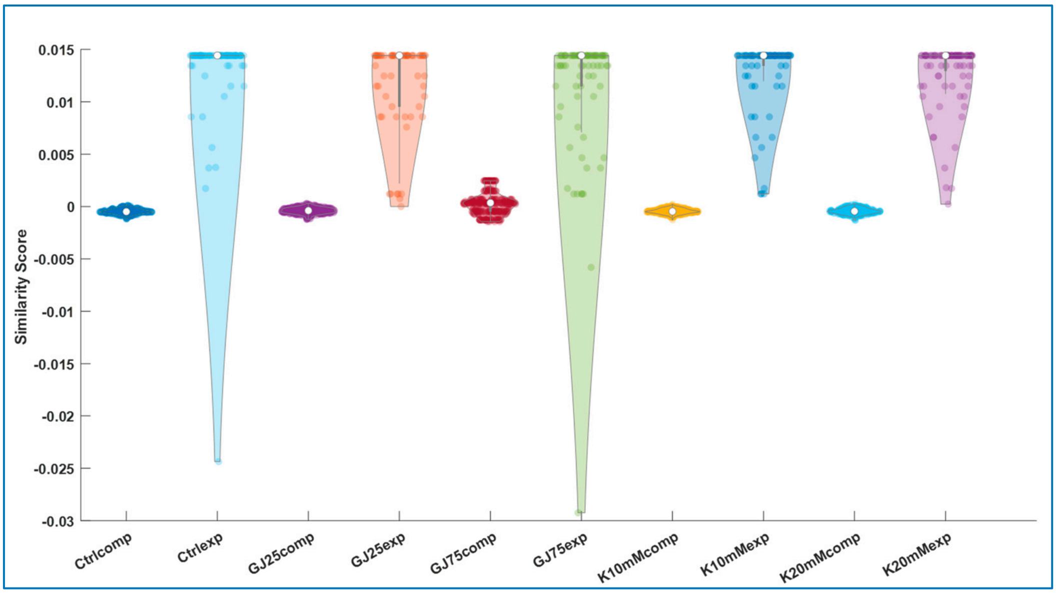

Figure S8. Violin plot of the similarity score distribution for each image set in comparison to the simulated 'GJ75comp' target pattern profile. Condition nomenclature: 'Ctrlcomp' = Un-modified culture media, simulated patterns; 'GJ25comp' = Weak inhibition of gap junction cell-cell communication, simulated patterns; 'GJ75comp' = Strong inhibition of gap junction cell-cell communication, simulated patterns; 'K10mMcomp' = 10 mM KCl culture media supplement, simulated patterns; 'K20mMcomp' = 20 mM KCl culture media supplement, simulated patterns; 'Ctrlexp' = Un-modified mTeSR culture media, experimentally-observed patterns; 'GJ25exp' = mTeSR culture media supplemented with 10 uM 18β-GA (gap junction inhibitor), experimentally-observed patterns; 'GJ75exp' = mTeSR culture media supplemented with 60 uM 18β-GA (gap junction inhibitor), experimentally-observed patterns; 'K10mMexp' = 10 mM KCl mTeSR culture media supplement, experimentally-observed patterns; 'K20mMexp' = 20 mM KCl mTeSR culture media supplement, experimentally-observed patterns.

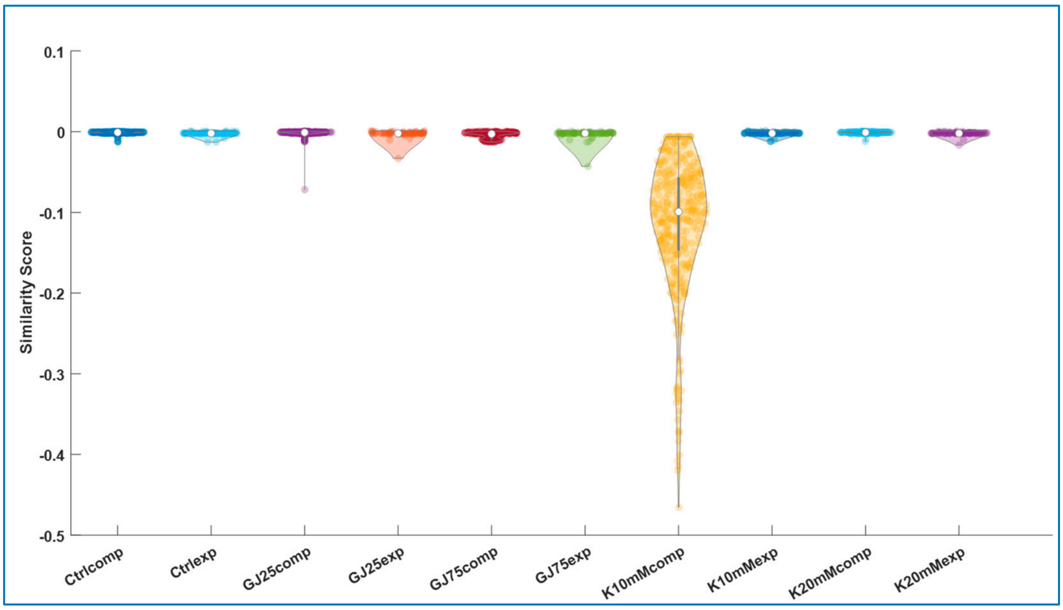

Figure S9. Violin plot of the similarity score distribution for each image set in comparison to the simulated 'K10mMcomp' target pattern profile. Condition nomenclature: 'Ctrlcomp' = Un-modified culture media, simulated patterns; 'GJ25comp' = Weak inhibition of gap junction cell-cell communication, simulated patterns; 'GJ75comp' = Strong inhibition of gap junction cell-cell communication, simulated patterns; 'K10mMcomp' = 10 mM KCl culture media supplement, simulated patterns; 'K20mMcomp' = 20 mM KCl culture media supplement, simulated patterns; 'Ctrlexp' = Un-modified mTeSR culture media, experimentally-observed patterns; 'GJ25exp' = mTeSR culture media supplemented with 10 uM 18β-GA (gap junction inhibitor), experimentally-observed patterns;

‘GJ75exp’ = mTeSR culture media supplemented with 60 uM 18β-GA (gap junction inhibitor), experimentally-observed patterns; ‘K10mMexp’ = 10 mM KCl mTeSR culture media supplement, experimentally-observed patterns; ‘K20mMexp’ = 20 mM KCl mTeSR culture media supplement, experimentally-observed patterns.

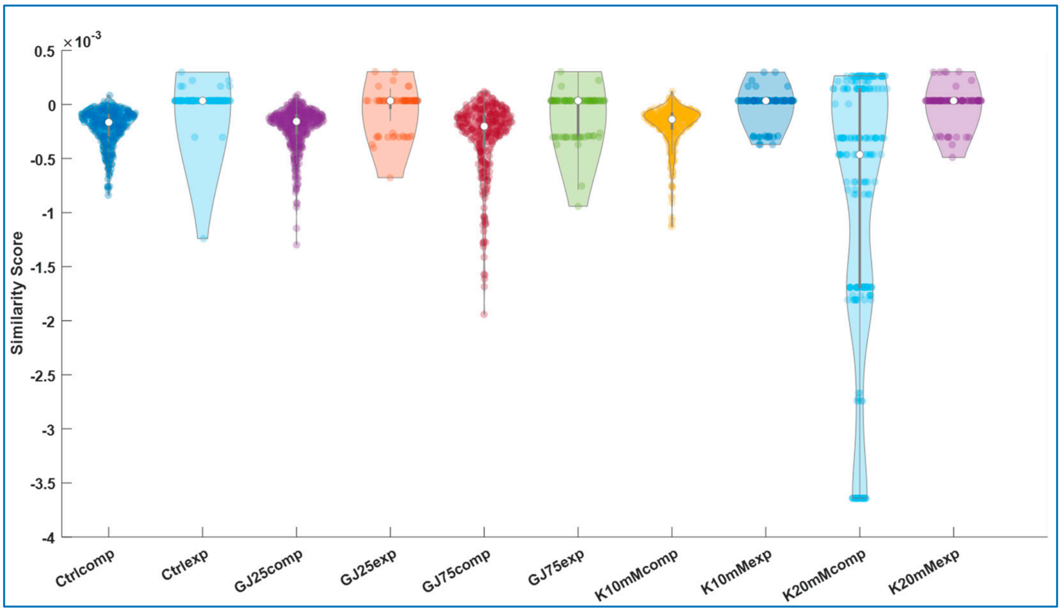

Figure S10. Violin plot of the similarity score distribution for each image set in comparison to the simulated ‘K20mMcomp’ target pattern profile. Condition nomenclature: ‘Ctrlcomp’ = Un-modified culture media, simulated patterns; ‘GJ25comp’ = Weak inhibition of gap junction cell-cell communication, simulated patterns; ‘GJ75comp’ = Strong inhibition of gap junction cell-cell communication, simulated patterns; ‘K10mMcomp’ = 10 mM KCl culture media supplement, simulated patterns; ‘K20mMcomp’ = 20 mM KCl culture media supplement, simulated patterns; ‘Ctrlexp’ = Un-modified mTeSR culture media, experimentally-observed patterns; ‘GJ25exp’ = mTeSR culture media supplemented with 10 uM 18β-GA (gap junction inhibitor), experimentally-observed patterns; ‘GJ75exp’ = mTeSR culture media supplemented with 60 uM 18β-GA (gap junction inhibitor), experimentally-observed patterns; ‘K10mMexp’ = 10 mM KCl mTeSR culture media supplement, experimentally-observed patterns; ‘K20mMexp’ = 20 mM KCl mTeSR culture media supplement, experimentally-observed patterns.

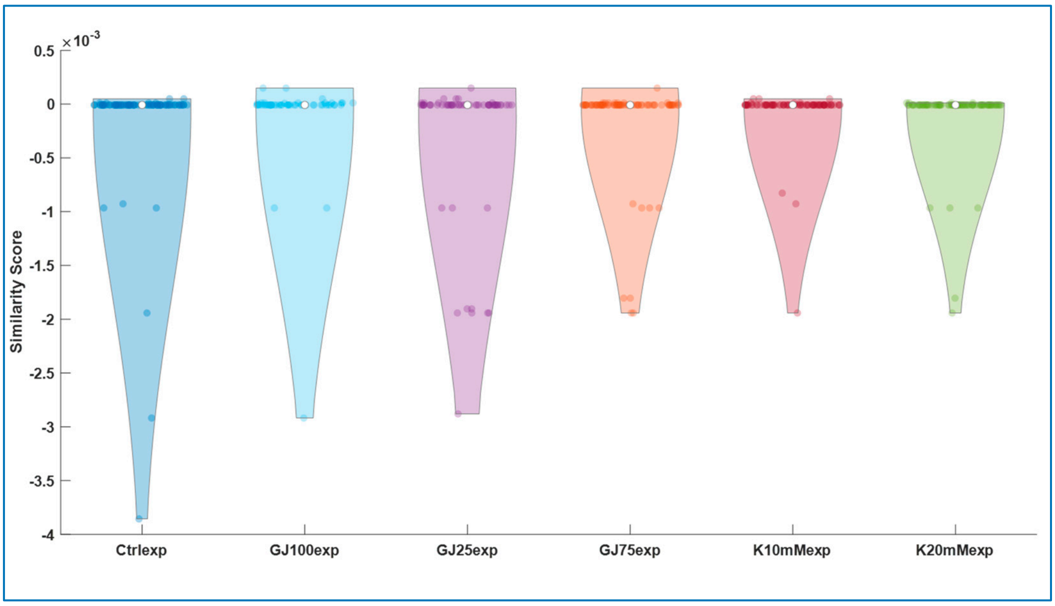

Figure S11. Violin plot of the similarity score distribution for each observed image set in comparison to the simulated ‘GJ100comp’ target pattern profile. Condition nomenclature: ‘Ctrlexp’ = Un-

modified mTeSR culture media, experimentally-observed patterns; 'GJ25exp' = mTeSR culture media supplemented with 10 uM 18β-GA (gap junction inhibitor), experimentally-observed patterns; 'GJ75exp' = mTeSR culture media supplemented with 60 uM 18β-GA (gap junction inhibitor), experimentally-observed patterns; 'GJ100exp' = mTeSR culture media supplemented with Cx43 (GJA1) expression knocked down, experimentally-observed patterns; 'K10mMexp' = 10 mM KCl mTeSR culture media supplement, experimentally-observed patterns; 'K20mMexp' = 20 mM KCl mTeSR culture media supplement, experimentally-observed patterns.

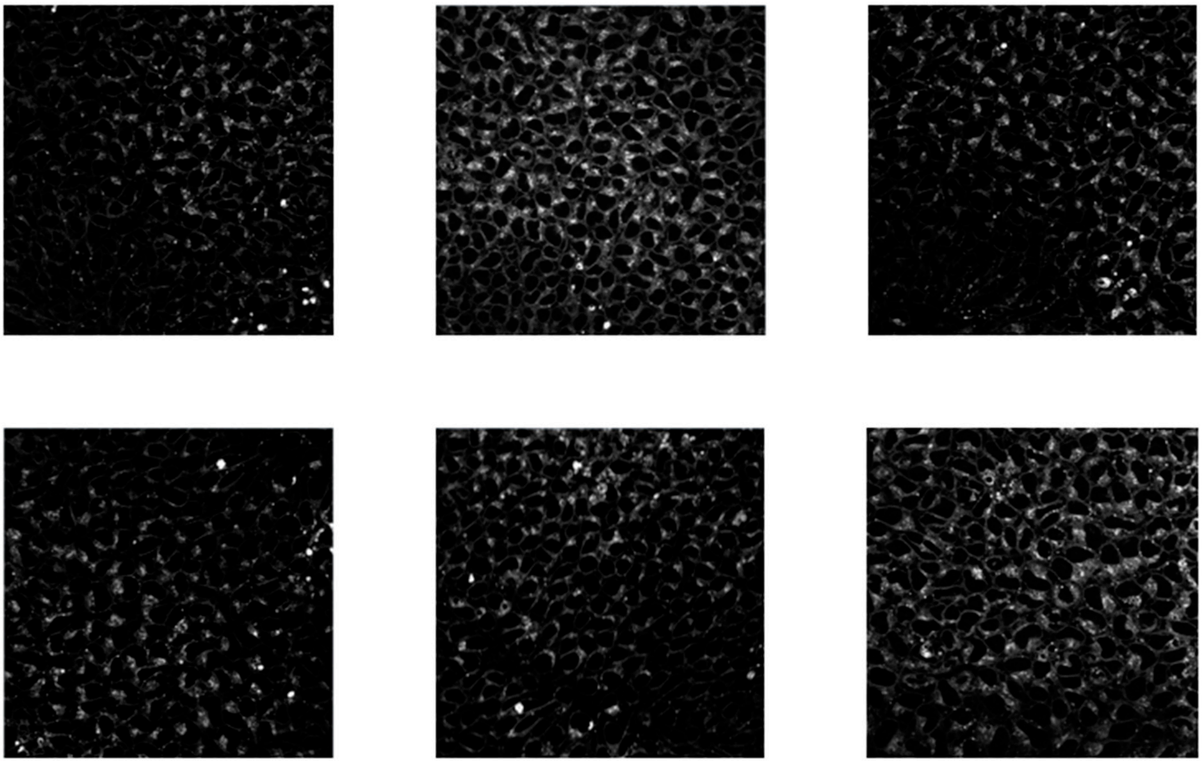

Figure S12. The raw output experiment DiBAC4[3] images of the 60 μM addition of β-GA culture condition.

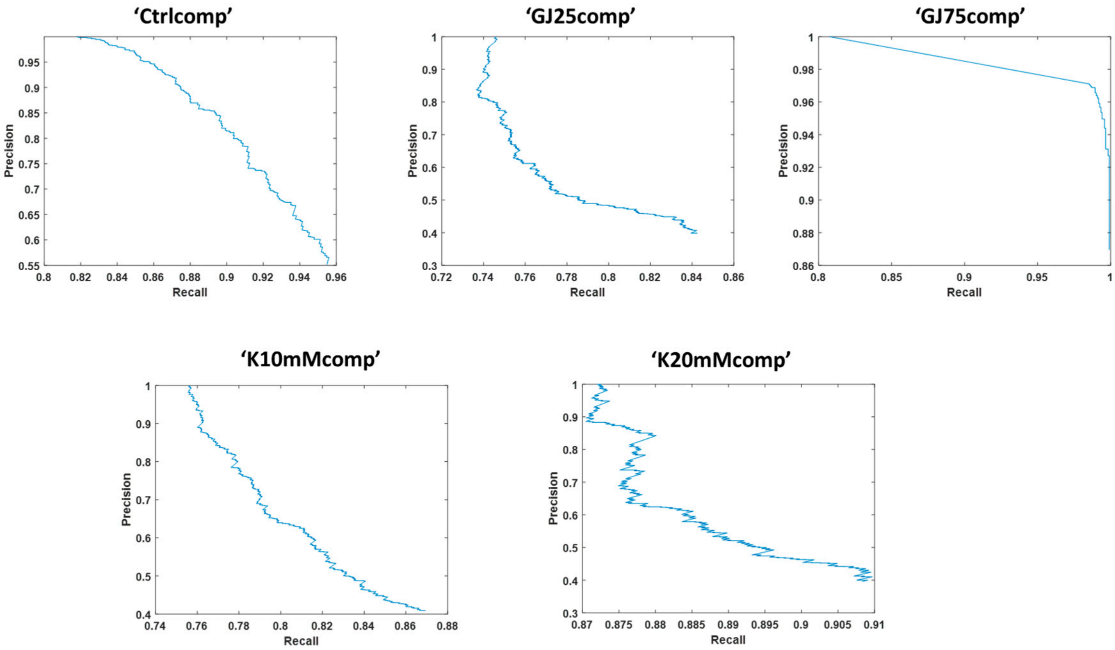

Figure S13. The precision-recall curves are shown for the five pattern classifiers defining the simulated culture conditions.
